# Supplementary material for: Relationships between consumption of ultra-processed foods, gestational weight gain and neonatal outcomes in a sample of US pregnant women
Source: PeerJ. 2017 Dec 7;5:e4091. doi: 10.7717/peerj.4091 (PMC5723430; doi:10.7717/peerj.4091)
Supplement: Appendix S1 — The appendix shows the 33 food subgroups within the four main NOVA groups, and explains why the ultra-processed subgroups were classified in this manner. [file peerj-05-4091-s001.docx]

The appendix shows the 33 food subgroups within the four main NOVA groups, and explains why the ultra-processed subgroups were classified in this manner.

| **Appendix: Classification of foods from DHQ II, along with justification.** | | | |
| --- | --- | --- | --- |
| **Classification** |  | **Subclassification** | **Justification for Classification (for ultra-processed foods only)** |
| Unprocessed or Minimally Processed Food | 1 | Meat (includes poultry) |  |
|  | 2 | Fruit |  |
|  | 3 | Milk |  |
|  | 4 | Grains |  |
|  | 5 | Roots and tubers |  |
|  | 6 | Eggs |  |
|  | 7 | Legumes |  |
|  | 8 | Fish and sea food |  |
|  | 9 | Vegetables |  |
|  | 10 | Other unprocessed or minimally processed foods |  |
| Processed Culinary Ingredients | 11 | Table sugar |  |
|  | 12 | Animal fats |  |
| Processed Foods | 13 | Cheese |  |
|  | 14 | Ham and other salted, smoked or canned meat or fish |  |
|  | 15 | Vegetables and other plant foods preserved in salt |  |
|  | 16 | Other processed foods |  |
| Ultra-processed Foods | 17 | Breads (stuffing, dressing, dumplings, bagels, English muffins, bread, dinner rolls, corn bread/muffins, biscuits) | In 1955, 94% of families bought bread, while only 5% baked bread. Most bought breads contain monoglycerides and high fructose corn syrup. |
|  | 18 | Cakes, cookies and pies (also includes pancakes, waffles, French toast, brownies, doughnuts, sweet rolls, Danish, pop-tarts, sweet muffins, dessert breads, fruit crisp/cobbler/strudel) | Most cakes, cookies and pies in American are bought in a ready to eat presentation at the store, and similar to bread only a small percentage of people bake such items from scratch. |
|  | 19 | Salty-snacks (potato chips, crackers, corn/tortilla chips, popcorn, pretzels) | Most popular popcorn brand (Orville Redenbacher) contains artificial flavors and TBHQ. |
|  | 20 | Frozen and shelf-stable plate meals (lasagna, stuffed shells, stuffed manicotti, ravioli, or tortellini, macaroni and cheese, beef stew/ pot pie, beef and noodles, beef and vegetables) | Most ready-to-eat meals contain mono- and diglycerides and/or modified food starch, both of which are chemically processed. |
|  | 21 | Soft drinks, carbonated | Soft drinks in the US are generally made from high fructose corn syrup. |
|  | 22 | Pizza (ready-to-eat/heat) | 74% of Americans never make pizza at home. Store-bought pizzas often contain preservatives, soy-protein isolates, and/or oil-based cheese substitutes. |
|  | 23 | Fruit drinks (sports drinks, energy drinks, applesauce, cranberry cocktail, Hi-C, lemonade, Kool-Aid) | All such drinks contain added sugar, and often have artificial sweeteners or colors. |
|  | 24 | Breakfast cereals | Seven of top 10 cereal brands contain artificial colors, preservatives, and/or high fructose corn syrup. |
|  | 25 | Sauces, dressings and gravies (mayonnaise, ketchup, gravy, salad dressing) | Major brands of ketchup (Heinz, Del Monte) contain high fructose corn syrup. 94.7% of households buy prepared salad dressing, and the most popular dressing (ranch) often contains xantham gum. |
|  | 26 | Reconstituted meat or fish products (bologna, salami, corned beef, pastrami, sausage) | These products often contain additives such as sodium erythorbate or BHT. |
|  | 27 | Ice cream and ice pops (frozen yogurt, sorbet, ices, ice cream, ice cream bars, sherbet) | Majority of ice cream brands contain additives such as xantham gum. |
|  | 28 | Sweet-snacks (energy/high-protein/breakfast bars, candy) | Such foods generally contain artificial flavors and sweeteners. |
|  | 29 | Milk drinks (chocolate milk, meal replacement/high protein beverages, yogurt) | Major brands of chocolate milk (TruMoo, Darigold, Purity) contain additives such as carrageenan or guar gum. Most yogurt is sweetened, and contains additives and/or high fructose corn syrup. |
|  | 30 | French fries and other potato products (also includes potato salad) | French fries and potato salad are generally store-bought, and often contain TBHQ, Calcium Disodium EDTA and/or xantham gum. |
|  | 31 | Sandwiches and hamburgers on bun (ready-to-eat/heat) | ‘Homemade’ sandwiches are generally made using store-bought bread, which is ultra-processed (see above). |
|  | 32 | Instant and canned soups | 64% of US households consume store-bought soup, which often contains MSG, modified food starch, and/or high fructose corn syrup. |
|  | 33 | Other ultra-processed foods (see list below) | Nondairy creamers generally contain high fructose corn syrup and/or partially hydrogenated oils. |
| Note: Other ultra-processed foods include syrup, artificial sweetener, nondairy creamer, fish sticks, fried fish, chicken salads/sandwiches/casseroles/stews, hot dogs, frankfurters, roast beef/steak sandwiches, pasta salad, macaroni salad, tacos, tostados, burritos, tamales, fajitas, enchiladas, quesadillas, and chimichangas, chili, liquor, mixed drinks, tofu, soy burgers, soy meat-substitutes, cold/iced tea. | | | |
