# Supplementary material for: Relationships between consumption of ultra-processed foods, gestational weight gain and neonatal outcomes in a sample of US pregnant women
Source: PeerJ. 2017 Dec 7;5:e4091. doi: 10.7717/peerj.4091 (PMC5723430; doi:10.7717/peerj.4091)
Supplement: Supplemental Information 3 — This is a blank copy of the questionnaire used in the study. [file peerj-05-4091-s011.pdf]

This is a sample form. Do not use for scanning.

NATIONAL INSTITUTES OF HEALTH

Diet History Questionnaire II

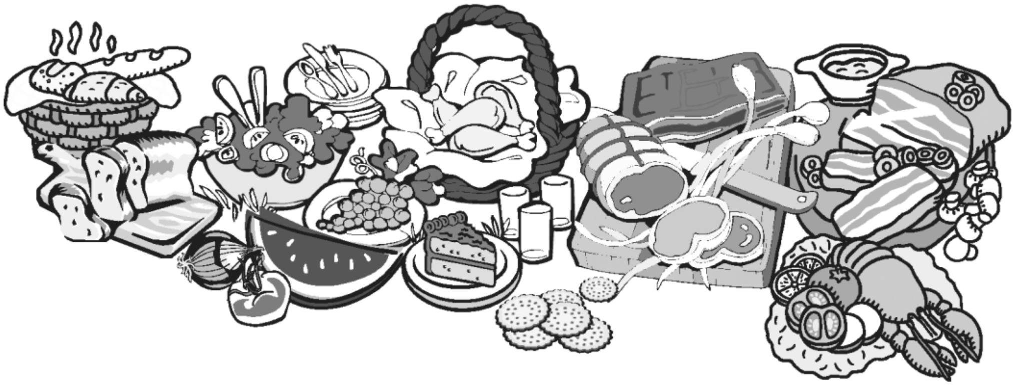

GENERAL INSTRUCTIONS

- **Answer each question as best you can. Estimate if you are not sure. A guess is better than leaving a blank.**
- **Use only a black ball-point pen. Do not use a pencil or felt-tip pen. Do not fold, staple, or tear the pages.**
- **Put an X in the box next to your answer.**
- **If you make any changes, cross out the incorrect answer and put an X in the box next to the correct answer. Also draw a circle around the correct answer.**
- **If you mark NEVER, NO, or DON'T KNOW for a question, please follow any arrows or instructions that direct you to the next question.**

BEFORE TURNING THE PAGE, PLEASE COMPLETE THE FOLLOWING QUESTIONS.

Today's date:

MONTH	DAY		YEAR
	|___|___|		
☐ Jan	☐0	☐0	☐ 2010
☐ Feb	☐1	☐1	☐ 2011
☐ Mar	☐2	☐2	☐ 2012
☐ Apr	☐3	☐3	☐ 2013
☐ May		☐4	☐ 2014
☐ Jun		☐5	☐ 2015
☐ Jul		☐6	☐ 2016
☐ Aug		☐7	☐ 2017
☐ Sep		☐8	☐ 2018
☐ Oct		☐9	☐ 2019
☐ Nov			☐ 2020
☐ Dec			

In what month were you born?

☐ Jan
☐ Feb
☐ Mar
☐ Apr
☐ May
☐ Jun
☐ Jul
☐ Aug
☐ Sep
☐ Oct
☐ Nov
☐ Dec

In what year were you born?

19 |___|___|

☐0	☐0
☐1	☐1
☐2	☐2
☐3	☐3
☐4	☐4
☐5	☐5
☐6	☐6
☐7	☐7
☐8	☐8
☐9	☐9

Are you male or female?

☐ Male
☐ Female

BAR CODE LABEL OR SUBJECT ID HERE

|___|___|___|___|___|___|___|___|

This is a sample form. Do not use for scanning.

1. Over the <u>past month</u>, how often did you drink **carrot juice**?

☐ NEVER (GO TO QUESTION 2)

☐ 1 time in past month
☐ 2–3 times in past month
☐ 1–2 times per week
☐ 3–4 times per week
☐ 5–6 times per week
☐ 1 time per day
☐ 2–3 times per day
☐ 4–5 times per day
☐ 6 or more times per day

1a. Each time you drank **carrot juice**, how much did you usually drink?

☐ Less than ½ cup (4 ounces)
☐ ½ to 1¼ cups (4 to 10 ounces)
☐ More than 1¼ cups (10 ounces)

2. Over the <u>past month</u>, how often did you drink **tomato juice** or **other vegetable juice**? *(Please do not include carrot juice.)*

☐ NEVER (GO TO QUESTION 3)

☐ 1 time in past month
☐ 2–3 times in past month
☐ 1–2 times per week
☐ 3–4 times per week
☐ 5–6 times per week
☐ 1 time per day
☐ 2–3 times per day
☐ 4–5 times per day
☐ 6 or more times per day

2a. Each time you drank **tomato juice** or **other vegetable juice**, how much did you usually drink?

☐ Less than ¾ cup (6 ounces)
☐ ¾ to 1¼ cups (6 to 10 ounces)
☐ More than 1¼ cups (10 ounces)

3. Over the <u>past month</u>, how often did you drink **orange juice** or **grapefruit juice?**

☐ NEVER (GO TO QUESTION 4)

☐ 1 time in past month
☐ 2–3 times in past month
☐ 1–2 times per week
☐ 3–4 times per week
☐ 5–6 times per week
☐ 1 time per day
☐ 2–3 times per day
☐ 4–5 times per day
☐ 6 or more times per day

3a. Each time you drank **orange juice** or **grapefruit juice**, how much did you usually drink?

☐ Less than ¾ cup (6 ounces)
☐ ¾ to 1¼ cups (6 to 10 ounces)
☐ More than 1¼ cups (10 ounces)

Question 4 appears in the next column

3b. How often was the orange juice or grapefruit juice you drank **calcium-fortified**?

☐ Almost never or never
☐ About ¼ of the time
☐ About ½ of the time
☐ About ¾ of the time
☐ Almost always or always

4. Over the <u>past month</u>, how often did you drink **other 100% fruit juice** or **100% fruit juice mixtures** (such as apple, grape, pineapple, or others)?

☐ NEVER (GO TO QUESTION 5)

☐ 1 time in past month
☐ 2–3 times in past month
☐ 1–2 times per week
☐ 3–4 times per week
☐ 5–6 times per week
☐ 1 time per day
☐ 2–3 times per day
☐ 4–5 times per day
☐ 6 or more times per day

4a. Each time you drank **other 100% fruit juice** or **100% fruit juice mixtures**, how much did you usually drink?

☐ Less than ¾ cup (6 ounces)
☐ ¾ to 1½ cups (6 to 12 ounces)
☐ More than 1½ cups (12 ounces)

4b. How often were the other 100% fruit juice or 100% fruit juice mixtures you drank **calcium-fortified**?

☐ Almost never or never
☐ About ¼ of the time
☐ About ½ of the time
☐ About ¾ of the time
☐ Almost always or always

5. How often did you drink **other fruit drinks** (such as cranberry cocktail, Hi-C, lemonade, or Kool-Aid, diet or regular)?

☐ NEVER (GO TO QUESTION 6)

☐ 1 time in past month
☐ 2–3 times in past month
☐ 1–2 times per week
☐ 3–4 times per week
☐ 5–6 times per week
☐ 1 time per day
☐ 2–3 times per day
☐ 4–5 times per day
☐ 6 or more times per day

Question 6 appears on the next page

This is a sample form. Do not use for scanning.

Over the past month…

5a. Each time you drank **fruit drinks**, how much did you usually drink?

- ☐ Less than 1 cup (8 ounces)
- ☐ 1 to 2 cups (8 to 16 ounces)
- ☐ More than 2 cups (16 ounces)

5b. How often were your fruit drinks **diet** or **sugar-free**?

- ☐ Almost never or never
- ☐ About ¼ of the time
- ☐ About ½ of the time
- ☐ About ¾ of the time
- ☐ Almost always or always

6. How often did you drink **milk as a beverage** (NOT in coffee, NOT in cereal)? *(Please do not include chocolate milk and hot chocolate.)*

- ☐ NEVER (GO TO QUESTION 7)

☐ 1 time in past month	☐ 1 time per day
☐ 2–3 times in past month	☐ 2–3 times per day
☐ 1–2 times per week	☐ 4–5 times per day
☐ 3–4 times per week	☐ 6 or more times per day
☐ 5–6 times per week	

6a. Each time you drank **milk as a beverage**, how much did you usually drink?

- ☐ Less than 1 cup (8 ounces)
- ☐ 1 to 1½ cups (8 to 12 ounces)
- ☐ More than 1½ cups (12 ounces)

6b. What kind of **milk** did you usually drink?

- ☐ Whole milk
- ☐ 2% fat milk
- ☐ 1 % fat milk
- ☐ Skim, nonfat, or ½% fat milk
- ☐ Soy milk
- ☐ Rice milk
- ☐ Other

7. How often did you drink **chocolate milk** (including hot chocolate)?

- ☐ NEVER (GO TO QUESTION 8)

☐ 1 time in past month	☐ 1 time per day
☐ 2–3 times in past month	☐ 2–3 times per day
☐ 1–2 times per week	☐ 4–5 times per day
☐ 3–4 times per week	☐ 6 or more times per day
☐ 5–6 times per week	

Question 8 appears in the next column

7a. Each time you drank **chocolate milk**, how much did you usually drink?

- ☐ Less than 1 cup (8 ounces)
- ☐ 1 to 1½ cups (8 to 12 ounces)
- ☐ More than 1½ cups (12 ounces)

7b. How often was the chocolate milk **reduced-fat** or **fat-free**?

- ☐ Almost never or never
- ☐ About ¼ of the time
- ☐ About ½ of the time
- ☐ About ¾ of the time
- ☐ Almost always or always

8. How often did you drink **meal replacement** or **high-protein beverages** (such as Instant Breakfast, Ensure, Slimfast, Sustacal or others)?

- ☐ NEVER (GO TO QUESTION 9)

☐ 1 time in past month	☐ 1 time per day
☐ 2–3 times in past month	☐ 2–3 times per day
☐ 1–2 times per week	☐ 4–5 times per day
☐ 3–4 times per week	☐ 6 or more times per day
☐ 5–6 times per week	

8a. Each time you drank **meal replacement** or **high-protein beverages**, how much did you usually drink?

- ☐ Less than 1 cup (8 ounces)
- ☐ 1 to 1½ cups (8 to 12 ounces)
- ☐ More than 1½ cups (12 ounces)

9. How often did you drink **soda** or **pop**?

- ☐ NEVER (GO TO QUESTION 10)

☐ 1 time in past month	☐ 1 time per day
☐ 2–3 times in past month	☐ 2–3 times per day
☐ 1–2 times per week	☐ 4–5 times per day
☐ 3–4 times per week	☐ 6 or more times per day
☐ 5–6 times per week	

9a. Each time you drank **soda** or **pop,** how much did you usually drink?

- ☐ Less than 12 ounces or less than 1 can or bottle
- ☐ 12 to 16 ounces or 1 can or bottle
- ☐ More than 16 ounces or more than 1 can or bottle

Question 10 appears on the next page

This is a sample form. Do not use for scanning.

Over the past month…

9b. How often were these sodas or pop **diet** or **sugar-free**?

- ☐ Almost never or never
- ☐ About ¼ of the time
- ☐ About ½ of the time
- ☐ About ¾ of the time
- ☐ Almost always or always

9c. How often were these sodas or pop **caffeine-free**?

- ☐ Almost never or never
- ☐ About ¼ of the time
- ☐ About ½ of the time
- ☐ About ¾ of the time
- ☐ Almost always or always

10. How often did you drink **sports drinks** (such as Propel, PowerAde, or Gatorade)?

- ☐ NEVER (GO TO QUESTION 11)
- ☐ 1 time in past month
- ☐ 2–3 times in past month
- ☐ 1–2 times per week
- ☐ 3–4 times per week
- ☐ 5–6 times per week
- ☐ 1 time per day
- ☐ 2–3 times per day
- ☐ 4–5 times per day
- ☐ 6 or more times per day

10a. Each time you drank **sports drinks**, how much did you usually drink?

- ☐ Less than 12 ounces or less than 1 bottle
- ☐ 12 to 24 ounces or 1 to 2 bottles
- ☐ More than 24 ounces or more than 2 bottles

11. How often did you drink **energy drinks** (such as Red Bull or Jolt)?

- ☐ NEVER (GO TO QUESTION 12)
- ☐ 1 time in past month
- ☐ 2–3 times in past month
- ☐ 1–2 times per week
- ☐ 3–4 times per week
- ☐ 5–6 times per week
- ☐ 1 time per day
- ☐ 2–3 times per day
- ☐ 4–5 times per day
- ☐ 6 or more times per day

11a. Each time you drank **energy drinks**, how much did you usually drink?

- ☐ Less than 8 ounces or less than 1 cup
- ☐ 8 to 16 ounces or 1 to 2 cups
- ☐ More than 16 ounces or more than 2 cups

Question 12 appears in the next column

12. How often did you drink **beer**?

- ☐ NEVER (GO TO QUESTION 13)
- ☐ 1 time in past month
- ☐ 2–3 times in past month
- ☐ 1–2 times per week
- ☐ 3–4 times per week
- ☐ 5–6 times per week
- ☐ 1 time per day
- ☐ 2–3 times per day
- ☐ 4–5 times per day
- ☐ 6 or more times per day

12a. Each time you drank **beer**, how much did you usually drink?

- ☐ Less than a 12-ounce can or bottle
- ☐ 1 to 3 12-ounce cans or bottles
- ☐ More than 3 12-ounce cans or bottles

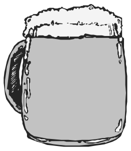

13. How often did you drink **water** (including tap, bottled, and carbonated water)?

- ☐ NEVER (GO TO QUESTION 14)
- ☐ 1 time in past month
- ☐ 2–3 times in past month
- ☐ 1–2 times per week
- ☐ 3–4 times per week
- ☐ 5–6 times per week
- ☐ 1 time per day
- ☐ 2–3 times per day
- ☐ 4–5 times per day
- ☐ 6 or more times per day

13a. Each time you drank **water**, how much did you usually drink?

- ☐ Less than 12 ounces or less than 1 bottle
- ☐ 12 to 24 ounces or 1 to 2 bottles
- ☐ More than 24 ounces or more than 2 bottles

13b. How often was the water you drank **tap water**?

- ☐ Almost never or never
- ☐ About ¼ of the time
- ☐ About ½ of the time
- ☐ About ¾ of the time
- ☐ Almost always or always

Question 14 appears on the next page

This is a sample form. Do not use for scanning.

Over the past month…

13c. How often was the water you drank **bottled, sweetened water** (with low or no-calorie sweetener, including carbonated water)?

- ☐ Almost never or never
- ☐ About ¼ of the time
- ☐ About ½ of the time
- ☐ About ¾ of the time
- ☐ Almost always or always

13d. How often was the water you drank **bottled, unsweetened water** (including carbonated water)?

- ☐ Almost never or never
- ☐ About ¼ of the time
- ☐ About ½ of the time
- ☐ About ¾ of the time
- ☐ Almost always or always

14. How often did you drink **wine** or **wine coolers**?

- ☐ NEVER (GO TO QUESTION 15)
- ☐ 1 time in past month
- ☐ 2–3 times in past month
- ☐ 1–2 times per week
- ☐ 3–4 times per week
- ☐ 5–6 times per week
- ☐ 1 time per day
- ☐ 2–3 times per day
- ☐ 4–5 times per day
- ☐ 6 or more times per day

14a. Each time you drank **wine** or **wine coolers**, how much did you usually drink?

- ☐ Less than 5 ounces or less than 1 glass
- ☐ 5 to 12 ounces or 1 to 2 glasses
- ☐ More than 12 ounces or more than 2 glasses

15. How often did you drink **liquor** or **mixed drinks**?

- ☐ NEVER (GO TO QUESTION 16)
- ☐ 1 time in past month
- ☐ 2–3 times in past month
- ☐ 1–2 times per week
- ☐ 3–4 times per week
- ☐ 5–6 times per week
- ☐ 1 time per day
- ☐ 2–3 times per day
- ☐ 4–5 times per day
- ☐ 6 or more times per day

15a. Each time you drank **liquor** or **mixed drinks**, how much did you usually drink?

- ☐ Less than 1 shot of liquor
- ☐ 1 to 3 shots of liquor
- ☐ More than 3 shots of liquor

Question 16 appears in the next column

16. How often did you eat **oatmeal, grits,** or **other cooked cereal?**

- ☐ NEVER (GO TO QUESTION 17)
- ☐ 1 time in past month
- ☐ 2–3 times in past month
- ☐ 1 time per week
- ☐ 2 times per week
- ☐ 3–4 times per week
- ☐ 5–6 times per week
- ☐ 1 time per day
- ☐ 2 or more times per day

16a. Each time you ate **oatmeal, grits,** or **other cooked cereal,** how much did you usually eat?

- ☐ Less than ¾ cup
- ☐ ¾ to 1¼ cups
- ☐ More than 1¼ cups

16b. How often was **butter** or **margarine** added to your oatmeal, grits or other cooked cereal?

- ☐ Almost never or never
- ☐ About ¼ of the time
- ☐ About ½ of the time
- ☐ About ¾ of the time
- ☐ Almost always or always

17. How often did you eat **cold cereal**?

- ☐ NEVER (GO TO QUESTION 18)
- ☐ 1 time in past month
- ☐ 2–3 times in past month
- ☐ 1 time per week
- ☐ 2 times per week
- ☐ 3–4 times per week
- ☐ 5–6 times per week
- ☐ 1 time per day
- ☐ 2 or more times per day

17a. Each time you ate **cold cereal**, how much did you usually eat?

- ☐ Less than 1 cup
- ☐ 1 to 2½ cups
- ☐ More than 2½ cups

17b. How often was the cold cereal you ate **Total Raisin Bran**, **Total Cereal**, or **Product 19**?

- ☐ Almost never or never
- ☐ About ¼ of the time
- ☐ About ½ of the time
- ☐ About ¾ of the time
- ☐ Almost always or always

Question 18 appears on the next page

This is a sample form. Do not use for scanning.

Over the past month…

17c. How often was the cold cereal you ate **All Bran**, **Fiber One**, **100% Bran**, or **All-Bran Bran Buds**?

- ☐ Almost never or never
- ☐ About ¼ of the time
- ☐ About ½ of the time
- ☐ About ¾ of the time
- ☐ Almost always or always

17d. How often was the cold cereal you ate **some other bran** or **fiber cereal** (such as Cheerios, Shredded Wheat, Raisin Bran, Bran Flakes, Grape-Nuts, Granola, Wheaties, or Healthy Choice)?

- ☐ Almost never or never
- ☐ About ¼ of the time
- ☐ About ½ of the time
- ☐ About ¾ of the time
- ☐ Almost always or always

17e. How often was the cold cereal you ate any **other type of cold cereal** (such as Corn Flakes, Rice Krispies, Frosted Flakes, Special K, Froot Loops, Cap'n Crunch, or others)?

- ☐ Almost never or never
- ☐ About ¼ of the time
- ☐ About ½ of the time
- ☐ About ¾ of the time
- ☐ Almost always or always

17f. Was **milk** added to your cold cereal?

- ☐ NO (GO TO QUESTION 18)
- ☐ YES

17g. What kind of **milk** was usually added?

- ☐ Whole milk
- ☐ 2% fat milk
- ☐ 1% fat milk
- ☐ Skim, nonfat, or ½% fat milk
- ☐ Soy milk
- ☐ Rice milk
- ☐ Other

17h. Each time **milk was added to your cold cereal**, how much was usually added?

- ☐ Less than ½ cup
- ☐ ½ to 1 cup
- ☐ More than 1 cup

Question 18 appears in the next column

18. How often did you eat **applesauce**?

- ☐ NEVER (GO TO QUESTION 19)

☐ 1 time in past month	☐ 3–4 times per week
☐ 2–3 times in past month	☐ 5–6 times per week
☐ 1 time per week	☐ 1 time per day
☐ 2 times per week	☐ 2 or more times per day

18a. Each time you ate **applesauce**, how much did you usually eat?

- ☐ Less than ½ cup
- ☐ ½ to 1 cup
- ☐ More than 1 cup

19. How often did you eat **apples**?

- ☐ NEVER (GO TO QUESTION 20)

☐ 1 time in past month	☐ 3–4 times per week
☐ 2–3 times in past month	☐ 5–6 times per week
☐ 1 time per week	☐ 1 time per day
☐ 2 times per week	☐ 2 or more times per day

19a. Each time you ate **apples**, how many did you usually eat?

- ☐ Less than 1 apple
- ☐ 1 apple
- ☐ More than 1 apple

20. How often did you eat **pears** (fresh, canned, or frozen)?

- ☐ NEVER (GO TO QUESTION 21)

☐ 1 time in past month	☐ 3–4 times per week
☐ 2–3 times in past month	☐ 5–6 times per week
☐ 1 time per week	☐ 1 time per day
☐ 2 times per week	☐ 2 or more times per day

20a. Each time you ate **pears**, how many did you usually eat?

- ☐ Less than 1 pear
- ☐ 1 pear
- ☐ More than 1 pear

21. How often did you eat **bananas?**

- ☐ NEVER (GO TO QUESTION 22)

☐ 1 time in past month	☐ 3–4 times per week
☐ 2–3 times in past month	☐ 5–6 times per week
☐ 1 time per week	☐ 1 time per day
☐ 2 times per week	☐ 2 or more times per day

Question 22 appears on the next page

This is a sample form. Do not use for scanning.

Over the past month…

21a. Each time you ate **bananas**, how many did you usually eat?

- ☐ Less than 1 banana
- ☐ 1 banana
- ☐ More than 1 banana

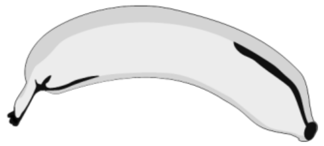

22. How often did you eat **dried fruit** (such as prunes or raisins)? *(Please do not include dried apricots.)*

☐ NEVER (GO TO QUESTION 23)

- ☐ 1 time in past month
- ☐ 2–3 times in past month
- ☐ 1 time per week
- ☐ 2 times per week
- ☐ 3–4 times per week
- ☐ 5–6 times per week
- ☐ 1 time per day
- ☐ 2 or more times per day

22a. Each time you ate **dried fruit**, how much did you usually eat?

- ☐ Less than 2 tablespoons
- ☐ 2 to 5 tablespoons
- ☐ More than 5 tablespoons

23. How often did you eat **peaches, nectarines,** or **plums**?

☐ NEVER (GO TO QUESTION 24)

- ☐ 1 time in past month
- ☐ 2–3 times in past month
- ☐ 1 time per week
- ☐ 2 times per week
- ☐ 3–4 times per week
- ☐ 5–6 times per week
- ☐ 1 time per day
- ☐ 2 or more times per day

23a. Each time you ate **peaches, nectarines,** or **plums,** how much did you usually eat?

- ☐ Less than 1 fruit or less than ½ cup
- ☐ 1 to 2 fruits or ½ to ¾ cup
- ☐ More than 2 fruits or more than ¾ cup

Question 24 appears in the next column

24. How often did you eat **grapes**?

☐ NEVER (GO TO QUESTION 25)

- ☐ 1 time in past month
- ☐ 2–3 times in past month
- ☐ 1 time per week
- ☐ 2 times per week
- ☐ 3–4 times per week
- ☐ 5–6 times per week
- ☐ 1 time per day
- ☐ 2 or more times per day

24a. Each time you ate **grapes**, how much did you usually eat?

- ☐ Less than ½ cup or less than 10 grapes
- ☐ ½ to 1 cup or 10 to 30 grapes
- ☐ More than 1 cup or more than 30 grapes

25. How often did you eat **cantaloupe**?

☐ NEVER (GO TO QUESTION 26)

- ☐ 1 time in past month
- ☐ 2–3 times in past month
- ☐ 1 time per week
- ☐ 2 times per week
- ☐ 3–4 times per week
- ☐ 5–6 times per week
- ☐ 1 time per day
- ☐ 2 or more times per day

25a. Each time you ate **cantaloupe**, how much did you usually eat?

- ☐ Less than ¼ melon or less than ½ cup
- ☐ ¼ melon or ½ to 1 cup
- ☐ More than ¼ melon or more than 1 cup

26. How often did you eat **melon, other than cantaloupe** (such as watermelon or honeydew)?

☐ NEVER (GO TO QUESTION 27)

- ☐ 1 time in past month
- ☐ 2–3 times in past month
- ☐ 1 time per week
- ☐ 2 times per week
- ☐ 3–4 times per week
- ☐ 5–6 times per week
- ☐ 1 time per day
- ☐ 2 or more times per day

26a. Each time you ate **melon other than cantaloupe**, how much did you usually eat?

- ☐ Less than ½ cup or 1 small wedge
- ☐ ½ to 2 cups or 1 medium wedge
- ☐ More than 2 cups or 1 large wedge

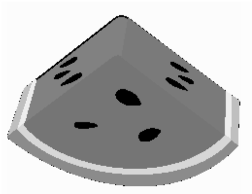

Question 27 appears on the next page

This is a sample form. Do not use for scanning.

Over the past month…

27. How often did you eat **strawberries**?

- ☐ NEVER (GO TO QUESTION 28)

- ☐ 1 time in past month
- ☐ 2–3 times in past month
- ☐ 1 time per week
- ☐ 2 times per week
- ☐ 3–4 times per week
- ☐ 5–6 times per week
- ☐ 1 time per day
- ☐ 2 or more times per day

27a. Each time you ate **strawberries**, how much did you usually eat?

- ☐ Less than ¼ cup or less than 3 berries
- ☐ ¼ to ¾ cup or 3 to 8 berries
- ☐ More than ¾ cup or more than 8 berries

28. How often did you eat **oranges, tangerines,** or **clementines**?

- ☐ NEVER (GO TO QUESTION 29)

- ☐ 1 time in past month
- ☐ 2–3 times in past month
- ☐ 1 time per week
- ☐ 2 times per week
- ☐ 3–4 times per week
- ☐ 5–6 times per week
- ☐ 1 time per day
- ☐ 2 or more times per day

28a. Each time you ate **oranges, tangerines,** or **clementines,** how many did you usually eat?

- ☐ Less than 1 fruit
- ☐ 1 fruit
- ☐ More than 1 fruit

29. How often did you eat **grapefruit**?

- ☐ NEVER (GO TO QUESTION 30)

- ☐ 1 time in past month
- ☐ 2–3 times in past month
- ☐ 1 time per week
- ☐ 2 times per week
- ☐ 3–4 times per week
- ☐ 5–6 times per week
- ☐ 1 time per day
- ☐ 2 or more times per day

29a. Each time you ate **grapefruit**, how much did you usually eat?

- ☐ Less than ½ grapefruit
- ☐ ½ grapefruit
- ☐ More than ½ grapefruit

Question 30 appears in the next column

30. How often did you eat **pineapple**?

- ☐ NEVER (GO TO QUESTION 31)

- ☐ 1 time in past month
- ☐ 2–3 times in past month
- ☐ 1 time per week
- ☐ 2 times per week
- ☐ 3–4 times per week
- ☐ 5–6 times per week
- ☐ 1 time per day
- ☐ 2 or more times per day

30a. Each time you ate **pineapple**, how much did you usually eat?

- ☐ Less than ¼ cup or less than 1 medium slice
- ☐ ¼ to ¾ cup or 1 medium slice
- ☐ More than ¾ cup or more than 1 medium slice

31. How often did you eat **other kinds of fruit**?

- ☐ NEVER (GO TO QUESTION 32)

- ☐ 1 time in past month
- ☐ 2–3 times in past month
- ☐ 1 time per week
- ☐ 2 times per week
- ☐ 3–4 times per week
- ☐ 5–6 times per week
- ☐ 1 time per day
- ☐ 2 or more times per day

31a. Each time you ate **other kinds of fruit**, how much did you usually eat?

- ☐ Less than ¼ cup
- ☐ ¼ to ¾ cup
- ☐ More than ¾ cup

32. How often did you eat **COOKED greens** (such as spinach, turnip, collard, mustard, chard, or kale)?

- ☐ NEVER (GO TO QUESTION 33)

- ☐ 1 time in past month
- ☐ 2–3 times in past month
- ☐ 1 time per week
- ☐ 2 times per week
- ☐ 3–4 times per week
- ☐ 5–6 times per week
- ☐ 1 time per day
- ☐ 2 or more times per day

32a. Each time you ate **COOKED greens**, how much did you usually eat?

- ☐ Less than ½ cup
- ☐ ½ to 1 cup
- ☐ More than 1 cup

33. How often did you eat **RAW greens** (such as spinach, turnip, collard, mustard, chard, or kale)? (*We will ask about lettuce later.)*

- ☐ NEVER (GO TO QUESTION 34)

- ☐ 1 time in past month
- ☐ 2–3 times in past month
- ☐ 1 time per week
- ☐ 2 times per week
- ☐ 3–4 times per week
- ☐ 5–6 times per week
- ☐ 1 time per day
- ☐ 2 or more times per day

Question 34 appears on the next page

This is a sample form. Do not use for scanning.

Over the past month…

33a. Each time you ate **RAW greens**, how much did you usually eat?

- ☐ Less than ½ cup
- ☐ ½ to 1 cup
- ☐ More than 1 cup

34. How often did you eat **coleslaw**?

- ☐ NEVER (GO TO QUESTION 35)
- ☐ 1 time in past month
- ☐ 2–3 times in past month
- ☐ 1 time per week
- ☐ 2 times per week
- ☐ 3–4 times per week
- ☐ 5–6 times per week
- ☐ 1 time per day
- ☐ 2 or more times per day

34a. Each time you ate **coleslaw**, how much did you usually eat?

- ☐ Less than ¼ cup
- ☐ ¼ to ¾ cup
- ☐ More than ¾ cup

35. How often did you eat **sauerkraut** or **cabbage** (other than coleslaw)?

- ☐ NEVER (GO TO QUESTION 36)
- ☐ 1 time in past month
- ☐ 2–3 times in past month
- ☐ 1 time per week
- ☐ 2 times per week
- ☐ 3–4 times per week
- ☐ 5–6 times per week
- ☐ 1 time per day
- ☐ 2 or more times per day

35a. Each time you ate **sauerkraut** or **cabbage**, how much did you usually eat?

- ☐ Less than ¼ cup
- ☐ ¼ to 1 cup
- ☐ More than 1 cup

36. How often did you eat **carrots** (fresh, canned, or frozen)?

- ☐ NEVER (GO TO QUESTION 37)
- ☐ 1 time in past month
- ☐ 2–3 times in past month
- ☐ 1 time per week
- ☐ 2 times per week
- ☐ 3–4 times per week
- ☐ 5–6 times per week
- ☐ 1 time per day
- ☐ 2 or more times per day

36a. Each time you ate **carrots**, how much did you usually eat?

- ☐ Less than ¼ cup or less than 2 baby carrots
- ☐ ¼ to ½ cup or 2 to 5 baby carrots
- ☐ More than ½ cup or more than 5 baby carrots

Question 37 appears in the next column

37. How often did you eat **string beans** or **green beans** (fresh, canned, or frozen)?

- ☐ NEVER (GO TO QUESTION 38)
- ☐ 1 time in past month
- ☐ 2–3 times in past month
- ☐ 1 time per week
- ☐ 2 times per week
- ☐ 3–4 times per week
- ☐ 5–6 times per week
- ☐ 1 time per day
- ☐ 2 or more times per day

37a. Each time you ate **string beans** or **green beans**, how much did you usually eat?

- ☐ Less than ½ cup
- ☐ ½ to 1 cup
- ☐ More than 1 cup

38. How often did you eat **peas** (fresh, canned, or frozen)?

- ☐ NEVER (GO TO QUESTION 39)
- ☐ 1 time in past month
- ☐ 2–3 times in past month
- ☐ 1 time per week
- ☐ 2 times per week
- ☐ 3–4 times per week
- ☐ 5–6 times per week
- ☐ 1 time per day
- ☐ 2 or more times per day

38a. Each time you ate **peas**, how much did you usually eat?

- ☐ Less than ¼ cup
- ☐ ¼ to ¾ cup
- ☐ More than ¾ cup

39. How often did you eat **corn**?

- ☐ NEVER (GO TO QUESTION 40)
- ☐ 1 time in past month
- ☐ 2–3 times in past month
- ☐ 1 time per week
- ☐ 2 times per week
- ☐ 3–4 times per week
- ☐ 5–6 times per week
- ☐ 1 time per day
- ☐ 2 or more times per day

39a. Each time you ate **corn**, how much did you usually eat?

- ☐ Less than 1 ear or less than ½ cup
- ☐ 1 ear or ½ to 1 cup
- ☐ More than 1 ear or more than 1 cup

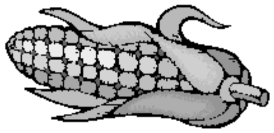

Question 40 appears on the next page

This is a sample form. Do not use for scanning.

Over the past month…

40. How often did you eat **broccoli** (fresh or frozen)?

- ☐ NEVER (GO TO QUESTION 41)
- ☐ 1 time in past month
- ☐ 2–3 times in past month
- ☐ 1 time per week
- ☐ 2 times per week
- ☐ 3–4 times per week
- ☐ 5–6 times per week
- ☐ 1 time per day
- ☐ 2 or more times per day

40a. Each time you ate **broccoli**, how much did you usually eat?

- ☐ Less than ¼ cup
- ☐ ¼ to 1 cup
- ☐ More than 1 cup

41. How often did you eat **cauliflower** or **Brussels sprouts** (fresh or frozen)?

- ☐ NEVER (GO TO QUESTION 42)
- ☐ 1 time in past month
- ☐ 2–3 times in past month
- ☐ 1 time per week
- ☐ 2 times per week
- ☐ 3–4 times per week
- ☐ 5–6 times per week
- ☐ 1 time per day
- ☐ 2 or more times per day

41a. Each time you ate **cauliflower** or **Brussels sprouts**, how much did you usually eat?

- ☐ Less than ¼ cup
- ☐ ¼ to ½ cup
- ☐ More than ½ cup

42. How often did you eat **asparagus** (fresh or frozen)?

- ☐ NEVER (GO TO QUESTION 43)
- ☐ 1 time in past month
- ☐ 2–3 times in past month
- ☐ 1 time per week
- ☐ 2 times per week
- ☐ 3–4 times per week
- ☐ 5–6 times per week
- ☐ 1 time per day
- ☐ 2 or more times per day

42a. Each time you ate **asparagus**, how much did you usually eat?

- ☐ Less than ⅓ cup or less than 4 spears
- ☐ ⅓ to ⅔ cup or 4 to 7 spears
- ☐ More than ⅔ cup or more than 7 spears

Question 43 appears in the next column

43. How often did you eat **winter squash** (such as pumpkin, butternut, or acorn)?

- ☐ NEVER (GO TO QUESTION 44)
- ☐ 1 time in past month
- ☐ 2–3 times in past month
- ☐ 1 time per week
- ☐ 2 times per week
- ☐ 3–4 times per week
- ☐ 5–6 times per week
- ☐ 1 time per day
- ☐ 2 or more times per day

43a. Each time you ate **winter squash**, how much did you usually eat?

- ☐ Less than ½ cup
- ☐ ½ to ¾ cup
- ☐ More than ¾ cup

44. How often did you eat **mixed vegetables**?

- ☐ NEVER (GO TO QUESTION 45)
- ☐ 1 time in past month
- ☐ 2–3 times in past month
- ☐ 1 time per week
- ☐ 2 times per week
- ☐ 3–4 times per week
- ☐ 5–6 times per week
- ☐ 1 time per day
- ☐ 2 or more times per day

44a. Each time you ate **mixed vegetables**, how much did you usually eat?

- ☐ Less than ½ cup
- ☐ ½ to 1 cup
- ☐ More than 1 cup

45. How often did you eat **onions**?

- ☐ NEVER (GO TO QUESTION 46)
- ☐ 1 time in past month
- ☐ 2–3 times in past month
- ☐ 1 time per week
- ☐ 2 times per week
- ☐ 3–4 times per week
- ☐ 5–6 times per week
- ☐ 1 time per day
- ☐ 2 or more times per day

45a. Each time you ate **onions**, how much did you usually eat?

- ☐ Less than 1 slice or less than 1 tablespoon
- ☐ 1 slice or 1 to 4 tablespoons
- ☐ More than 1 slice or more than 4 tablespoons

Question 46 appears on the next page

This is a sample form. Do not use for scanning.

Over the past month…

46. Now think about all the **cooked vegetables** you ate in the past month and how they were prepared. How often were your vegetables **COOKED WITH** some sort of **fat**, including oil spray? *(Please do not include potatoes.)*

- ☐ NEVER (GO TO QUESTION 47)
- ☐ 1 time in past month
- ☐ 2–3 times in past month
- ☐ 1 time per week
- ☐ 2 times per week
- ☐ 3–4 times per week
- ☐ 5–6 times per week
- ☐ 1 time per day
- ☐ 2 or more times per day

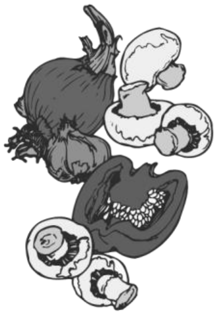

46a. Which fats were usually added to your vegetables **DURING COOKING**? *(Please do not include potatoes.* ***Mark all that apply.****)*

- ☐ Margarine (including low-fat)
- ☐ Butter (including low-fat)
- ☐ Lard, fatback, or bacon fat
- ☐ Olive oil
- ☐ Corn oil
- ☐ Canola or rapeseed oil
- ☐ Oil spray, such as Pam or others
- ☐ Other kinds of oils
- ☐ None of the above

47. Now, thinking again about all the **cooked vegetables** you ate in the past month, how often was some sort of fat, sauce, or dressing added **AFTER COOKING OR AT THE TABLE**? *(Please do not include potatoes.)*

- ☐ NEVER (GO TO QUESTION 48)
- ☐ 1 time in past month
- ☐ 2–3 times in past month
- ☐ 1–2 times per week
- ☐ 3–4 times per week
- ☐ 5–6 times per week
- ☐ 1 time per day
- ☐ 2 times per day
- ☐ 3 or more times per day

Question 48 appears in the next column

47a. Which fats, sauces, or dressings were usually added **AFTER COOKING OR AT THE TABLE**? *(Please do not include potatoes.* ***Mark all that apply.****)*

- ☐ Margarine (including low-fat)
- ☐ Butter (including low-fat)
- ☐ Lard, fatback, or bacon fat
- ☐ Salad dressing
- ☐ Cheese sauce
- ☐ White sauce
- ☐ Other

47b. If margarine, butter, lard, fatback, or bacon fat was added to your cooked vegetables **AFTER COOKING OR AT THE TABLE**, how much did you usually add?

- ☐ Did not usually add these
- ☐ Less than 1 teaspoon
- ☐ 1 to 3 teaspoons
- ☐ More than 3 teaspoons

47c. If salad dressing, cheese sauce, or white sauce was added to your cooked vegetables **AFTER COOKING OR AT THE TABLE**, how much did you usually add?

- ☐ Did not usually add these
- ☐ Less than 1 tablespoon
- ☐ 1 to 3 tablespoons
- ☐ More than 3 tablespoons

48. How often did you eat **sweet peppers** (green, red, or yellow)?

- ☐ NEVER (GO TO QUESTION 49)
- ☐ 1 time in past month
- ☐ 2–3 times in past month
- ☐ 1 time per week
- ☐ 2 times per week
- ☐ 3–4 times per week
- ☐ 5–6 times per week
- ☐ 1 time per day
- ☐ 2 or more times per day

48a. Each time you ate **sweet peppers**, how much did you usually eat?

- ☐ Less than ⅛ pepper
- ☐ ⅛ to ¼ pepper
- ☐ More than ¼ pepper

Question 49 appears on the next page

This is a sample form. Do not use for scanning.

Over the past month…

49. How often did you eat **fresh tomatoes** (including those in salads)?

- ☐ NEVER (GO TO QUESTION 50)
- ☐ 1 time in past month
- ☐ 2–3 times in past month
- ☐ 1 time per week
- ☐ 2 times per week
- ☐ 3–4 times per week
- ☐ 5–6 times per week
- ☐ 1 time per day
- ☐ 2 or more times per day

49a. Each time you ate **fresh tomatoes**, how much did you usually eat?

- ☐ Less than ¼ tomato
- ☐ ¼ to ½ tomato
- ☐ More than ½ tomato

50. How often did you eat **lettuce salads** (with or without other vegetables)?

- ☐ NEVER (GO TO QUESTION 51)
- ☐ 1 time in past month
- ☐ 2–3 times in past month
- ☐ 1 time per week
- ☐ 2 times per week
- ☐ 3–4 times per week
- ☐ 5–6 times per week
- ☐ 1 time per day
- ☐ 2 or more times per day

50a. Each time you ate **lettuce salads**, how much did you usually eat?

- ☐ Less than ¼ cup
- ☐ ¼ to 1¼ cups
- ☐ More than 1¼ cups

50b. How often did the lettuce salads you ate include **dark green lettuce**?

- ☐ Almost never or never
- ☐ About ¼ of the time
- ☐ About ½ of the time
- ☐ About ¾ of the time
- ☐ Almost always or always

51. How often did you eat **salad dressing** (including low-fat) **on salads**?

- ☐ NEVER (GO TO QUESTION 52)
- ☐ 1 time in past month
- ☐ 2–3 times in past month
- ☐ 1 time per week
- ☐ 2 times per week
- ☐ 3–4 times per week
- ☐ 5–6 times per week
- ☐ 1 time per day
- ☐ 2 or more times per day

51a. Each time you ate **salad dressing on salads**, how much did you usually eat?

- ☐ Less than 2 tablespoons
- ☐ 2 to 4 tablespoons
- ☐ More than 4 tablespoons

Question 52 appears in the next column

52. How often did you eat **sweet potatoes** or **yams**?

- ☐ NEVER (GO TO QUESTION 53)
- ☐ 1 time in past month
- ☐ 2–3 times in past month
- ☐ 1 time per week
- ☐ 2 times per week
- ☐ 3–4 times per week
- ☐ 5–6 times per week
- ☐ 1 time per day
- ☐ 2 or more times per day

52a. Each time you ate **sweet potatoes** or **yams**, how much did you usually eat?

- ☐ 1 small potato or less than ¼ cup
- ☐ 1 medium potato or ¼ to ¾ cup
- ☐ 1 large potato or more than ¾ cup

53. How often did you eat **French fries**, **home fries**, **hash browned potatoes**, or **tater tots**?

- ☐ NEVER (GO TO QUESTION 54)
- ☐ 1 time in past month
- ☐ 2–3 times in past month
- ☐ 1 time per week
- ☐ 2 times per week
- ☐ 3–4 times per week
- ☐ 5–6 times per week
- ☐ 1 time per day
- ☐ 2 or more times per day

53a. Each time you ate **French fries**, **home fries**, **hash browned potatoes**, or **tater tots** how much did you usually eat?

- ☐ Less than 10 fries or less than ½ cup
- ☐ 10 to 25 fries or ½ to 1 cup
- ☐ More than 25 fries or more than 1 cup

54. How often did you eat **potato salad**?

- ☐ NEVER (GO TO QUESTION 55)
- ☐ 1 time in past month
- ☐ 2–3 times in past month
- ☐ 1 time per week
- ☐ 2 times per week
- ☐ 3–4 times per week
- ☐ 5–6 times per week
- ☐ 1 time per day
- ☐ 2 or more times per day

54a. Each time you ate **potato salad**, how much did you usually eat?

- ☐ Less than ½ cup
- ☐ ½ to 1 cup
- ☐ More than 1 cup

55. How often did you eat **baked, boiled,** or **mashed potatoes**?

- ☐ NEVER (GO TO QUESTION 56)
- ☐ 1 time in past month
- ☐ 2–3 times in past month
- ☐ 1 time per week
- ☐ 2 times per week
- ☐ 3–4 times per week
- ☐ 5–6 times per week
- ☐ 1 time per day
- ☐ 2 or more times per day

Question 56 appears on the next page

This is a sample form. Do not use for scanning.

Over the past month…

55a. Each time you ate **baked, boiled,** or **mashed potatoes**, how much did you usually eat?

- ☐ 1 small potato or less than ½ cup
- ☐ 1 medium potato or ½ to 1 cup
- ☐ 1 large potato or more than 1 cup

55b. How often was **sour cream** (including low-fat) added to your potatoes, **EITHER IN COOKING OR AT THE TABLE**?

- ☐ Almost never or never (GO TO QUESTION 55d)
- ☐ About ¼ of the time
- ☐ About ½ of the time
- ☐ About ¾ of the time
- ☐ Almost always or always

55c. Each time **sour cream** was added to your potatoes, how much was usually added?

- ☐ Less than 1 tablespoon
- ☐ 1 to 3 tablespoons
- ☐ More than 3 tablespoons

55d. How often was **margarine** (including low-fat) added to your potatoes, **EITHER IN COOKING OR AT THE TABLE**?

- ☐ Almost never or never
- ☐ About ¼ of the time
- ☐ About ½ of the time
- ☐ About ¾ of the time
- ☐ Almost always or always

55e. How often was **butter** (including low-fat) added to your potatoes, **EITHER IN COOKING OR AT THE TABLE**?

- ☐ Almost never or never
- ☐ About ¼ of the time
- ☐ About ½ of the time
- ☐ About ¾ of the time
- ☐ Almost always or always

55f. Each time **margarine** or **butter** was added to your potatoes, how much was usually added?

- ☐ Never added
- ☐ Less than 1 teaspoon
- ☐ 1 to 3 teaspoons
- ☐ More than 3 teaspoons

Question 56 appears in the next column

55g. How often was **cheese** or **cheese sauce** added to your potatoes, **EITHER IN COOKING OR AT THE TABLE**?

- ☐ Almost never or never (GO TO QUESTION 56)
- ☐ About ¼ of the time
- ☐ About ½ of the time
- ☐ About ¾ of the time
- ☐ Almost always or always

55h. Each time **cheese** or **cheese sauce** was added to your potatoes, how much was usually added?

- ☐ Less than 1 tablespoon
- ☐ 1 to 3 tablespoons
- ☐ More than 3 tablespoons

56. How often did you eat **salsa**?

- ☐ NEVER (GO TO QUESTION 57)

☐ 1 time in past month	☐ 3–4 times per week
☐ 2–3 times in past month	☐ 5–6 times per week
☐ 1 time per week	☐ 1 time per day
☐ 2 times per week	☐ 2 or more times per day

56a. Each time you ate **salsa**, how much did you usually eat?

- ☐ Less than 1 tablespoon
- ☐ 1 to 5 tablespoons
- ☐ More than 5 tablespoons

57. How often did you eat **catsup**?

- ☐ NEVER (GO TO QUESTION 58)

☐ 1 time in past month	☐ 3–4 times per week
☐ 2–3 times in past month	☐ 5–6 times per week
☐ 1 time per week	☐ 1 time per day
☐ 2 times per week	☐ 2 or more times per day

57a. Each time you ate **catsup**, how much did you usually eat?

- ☐ Less than 1 teaspoon
- ☐ 1 to 6 teaspoons
- ☐ More than 6 teaspoons

58. How often did you eat **stuffing, dressing,** or **dumplings**?

- ☐ NEVER (GO TO QUESTION 59)

☐ 1 time in past month	☐ 3–4 times per week
☐ 2–3 times in past month	☐ 5–6 times per week
☐ 1 time per week	☐ 1 time per day
☐ 2 times per week	☐ 2 or more times per day

Question 59 appears on the next page

This is a sample form. Do not use for scanning.

Over the past month…

58a. Each time you ate **stuffing, dressing,** or **dumplings**, how much did you usually eat?

- ☐ Less than ½ cup
- ☐ ½ to 1 cup
- ☐ More than 1 cup

59. How often did you eat **chili**?

- ☐ NEVER (GO TO QUESTION 60)
- ☐ 1 time in past month
- ☐ 2–3 times in past month
- ☐ 1 time per week
- ☐ 2 times per week
- ☐ 3–4 times per week
- ☐ 5–6 times per week
- ☐ 1 time per day
- ☐ 2 or more times per day

59a. Each time you ate **chili**, how much did you usually eat?

- ☐ Less than ½ cup
- ☐ ½ to 1¾ cups
- ☐ More than 1¾ cups

60. How often did you eat **Mexican foods** (such as tacos, tostados, burritos, tamales, fajitas, enchiladas, quesadillas, and chimichangas)?

- ☐ NEVER (GO TO QUESTION 61)
- ☐ 1 time in past month
- ☐ 2–3 times in past month
- ☐ 1 time per week
- ☐ 2 times per week
- ☐ 3–4 times per week
- ☐ 5–6 times per week
- ☐ 1 time per day
- ☐ 2 or more times per day

60a. Each time you ate **Mexican foods**, how much did you usually eat?

- ☐ Less than 1 taco, burrito, etc.
- ☐ 1 to 2 tacos, burritos, etc.
- ☐ More than 2 tacos, burritos, etc.

61. How often did you eat **cooked dried beans** (such as baked beans, pintos, kidney, blackeyed peas, lima, lentils, soybeans, or refried beans)? *(Please do not include bean soups or chili.)*

- ☐ NEVER (GO TO QUESTION 62)
- ☐ 1 time in past month
- ☐ 2–3 times in past month
- ☐ 1 time per week
- ☐ 2 times per week
- ☐ 3–4 times per week
- ☐ 5–6 times per week
- ☐ 1 time per day
- ☐ 2 or more times per day

61a. Each time you ate **beans**, how much did you usually eat?

- ☐ Less than ½ cup
- ☐ ½ to 1 cup
- ☐ More than 1 cup

Question 62 appears in the next column

61b. How often were the beans you ate **refried beans**, **beans prepared with any type of fat**, or **with meat added**?

- ☐ Almost never or never
- ☐ About ¼ of the time
- ☐ About ½ of the time
- ☐ About ¾ of the time
- ☐ Almost always or always

62. How often did you eat **other kinds of vegetables**?

- ☐ NEVER (GO TO QUESTION 63)
- ☐ 1 time in past month
- ☐ 2–3 times in past month
- ☐ 1 time per week
- ☐ 2 times per week
- ☐ 3–4 times per week
- ☐ 5–6 times per week
- ☐ 1 time per day
- ☐ 2 or more times per day

62a. Each time you ate **other kinds of vegetables**, how much did you usually eat?

- ☐ Less than ¼ cup
- ☐ ¼ to ½ cup
- ☐ More than ½ cup

63. How often did you eat **rice** or **other cooked grains** (such as bulgur, cracked wheat, or millet)?

- ☐ NEVER (GO TO QUESTION 64)
- ☐ 1 time in past month
- ☐ 2–3 times in past month
- ☐ 1 time per week
- ☐ 2 times per week
- ☐ 3–4 times per week
- ☐ 5–6 times per week
- ☐ 1 time per day
- ☐ 2 or more times per day

63a. Each time you ate **rice** or **other cooked grains**, how much did you usually eat?

- ☐ Less than ½ cup
- ☐ ½ to 1½ cups
- ☐ More than 1½ cups

63b. How often was **butter**, **margarine**, or **oil** added to your rice or other cooked grains **IN COOKING OR AT THE TABLE**?

- ☐ Almost never or never
- ☐ About ¼ of the time
- ☐ About ½ of the time
- ☐ About ¾ of the time
- ☐ Almost always or always

Question 64 appears on the next page

This is a sample form. Do not use for scanning.

Over the past month…

64. How often did you eat **pancakes, waffles,** or **French toast**?

☐ NEVER (GO TO QUESTION 65)

☐ 1 time in past month
☐ 2–3 times in past month
☐ 1 time per week
☐ 2 times per week
☐ 3–4 times per week
☐ 5–6 times per week
☐ 1 time per day
☐ 2 or more times per day

64a. Each time you ate **pancakes, waffles,** or **French toast**, how much did you usually eat?

☐ Less than 1 medium piece
☐ 1 to 3 medium pieces
☐ More than 3 medium pieces

64b. How often was **margarine** (including low-fat) added to your pancakes, waffles, or French toast **AFTER COOKING OR AT THE TABLE**?

☐ Almost never or never
☐ About ¼ of the time
☐ About ½ of the time
☐ About ¾ of the time
☐ Almost always or always

64c. How often was **butter** (including low-fat) added to your pancakes, waffles, or French toast **AFTER COOKING OR AT THE TABLE**?

☐ Almost never or never
☐ About ¼ of the time
☐ About ½ of the time
☐ About ¾ of the time
☐ Almost always or always

64d. Each time **margarine** or **butter** was added to your pancakes, waffles, or French toast, how much was usually added?

☐ Never added
☐ Less than 1 teaspoon
☐ 1 to 3 teaspoons
☐ More than 3 teaspoons

64e. How often was **syrup** added to your pancakes, waffles, or French toast?

☐ Almost never or never (GO TO QUESTION 65)
☐ About ¼ of the time
☐ About ½ of the time
☐ About ¾ of the time
☐ Almost always or always

Question 65 appears in the next column

64f. Each time **syrup** was added to your pancakes, waffles, or French toast, how much was usually added?

☐ Less than 1 tablespoon
☐ 1 to 4 tablespoons
☐ More than 4 tablespoons

65. How often did you eat **lasagna, stuffed shells, stuffed manicotti, ravioli,** or **tortellini**? *(Please do not include spaghetti or other pasta.)*

☐ NEVER (GO TO QUESTION 66)

☐ 1 time in past month
☐ 2–3 times in past month
☐ 1 time per week
☐ 2 times per week
☐ 3–4 times per week
☐ 5–6 times per week
☐ 1 time per day
☐ 2 or more times per day

65a. Each time you ate **lasagna**, **stuffed shells**, **stuffed manicotti**, **ravioli**, or **tortellini**, how much did you usually eat?

☐ Less than 1 cup
☐ 1 to 2 cups
☐ More than 2 cups

66. How often did you eat **macaroni and cheese**?

☐ NEVER (GO TO QUESTION 67)

☐ 1 time in past month
☐ 2–3 times in past month
☐ 1 time per week
☐ 2 times per week
☐ 3–4 times per week
☐ 5–6 times per week
☐ 1 time per day
☐ 2 or more times per day

66a. Each time you ate **macaroni and cheese**, how much did you usually eat?

☐ Less than 1 cup
☐ 1 to 1½ cups
☐ More than 1½ cups

67. How often did you eat **pasta salad** or **macaroni salad**?

☐ NEVER (GO TO QUESTION 68)

☐ 1 time in past month
☐ 2–3 times in past month
☐ 1 time per week
☐ 2 times per week
☐ 3–4 times per week
☐ 5–6 times per week
☐ 1 time per day
☐ 2 or more times per day

Question 68 appears on the next page

This is a sample form. Do not use for scanning.

Over the <u>past month</u>…

67a. Each time you ate **pasta salad** or **macaroni salad**, how much did you usually eat?

- ☐ Less than ½ cup
- ☐ ½ to 1 cup
- ☐ More than 1 cup

68. Other than the pastas listed in Questions 65, 66, and 67, how often did you eat **pasta, spaghetti**, or **other noodles**?

- ☐ NEVER (GO TO QUESTION 69)
- ☐ 1 time in past month
- ☐ 2–3 times in past month
- ☐ 1 time per week
- ☐ 2 times per week
- ☐ 3–4 times per week
- ☐ 5–6 times per week
- ☐ 1 time per day
- ☐ 2 or more times per day

68a. Each time you ate **pasta, spaghetti, or other noodles**, how much did you usually eat?

- ☐ Less than 1 cup
- ☐ 1 to 3 cups
- ☐ More than 3 cups

68b. How often did you eat your pasta, spaghetti, or other noodles with **tomato sauce** or **spaghetti sauce made WITH meat**?

- ☐ Almost never or never
- ☐ About ¼ of the time
- ☐ About ½ of the time
- ☐ About ¾ of the time
- ☐ Almost always or always

68c. How often did you eat your pasta, spaghetti, or other noodles with **tomato sauce** or **spaghetti sauce made WITHOUT meat**?

- ☐ Almost never or never
- ☐ About ¼ of the time
- ☐ About ½ of the time
- ☐ About ¾ of the time
- ☐ Almost always or always

68d. How often did you eat your pasta, spaghetti, or other noodles with **margarine, butter, oil,** or **cream sauce**?

- ☐ Almost never or never
- ☐ About ¼ of the time
- ☐ About ½ of the time
- ☐ About ¾ of the time
- ☐ Almost always or always

Question 69 appears in the next column

69. How often did you eat **bagels** or **English muffins**?

- ☐ NEVER (GO TO INTRODUCTION TO QUESTION 70)
- ☐ 1 time in past month
- ☐ 2–3 times in past month
- ☐ 1 time per week
- ☐ 2 times per week
- ☐ 3–4 times per week
- ☐ 5–6 times per week
- ☐ 1 time per day
- ☐ 2 or more times per day

69a. How often were the bagels or English muffins you ate **whole wheat**?

- ☐ Almost never or never
- ☐ About ¼ of the time
- ☐ About ½ of the time
- ☐ About ¾ of the time
- ☐ Almost always or always

69b. Each time you ate **bagels** or **English muffins**, how many did you usually eat?

- ☐ Less than 1 bagel or English muffin
- ☐ 1 bagel or English muffin
- ☐ More than 1 bagel or English muffin

69c. How often was **margarine** (including low-fat) added to your bagels or English muffins?

- ☐ Almost never or never
- ☐ About ¼ of the time
- ☐ About ½ of the time
- ☐ About ¾ of the time
- ☐ Almost always or always

69d. How often was **butter** (including low-fat) added to your bagels or English muffins?

- ☐ Almost never or never
- ☐ About ¼ of the time
- ☐ About ½ of the time
- ☐ About ¾ of the time
- ☐ Almost always or always

69e. Each time **margarine** or **butter** was added to your bagels or English muffins, how much was usually added?

- ☐ Never added
- ☐ Less than 1 teaspoon
- ☐ 1 to 2 teaspoons
- ☐ More than 2 teaspoons

Introduction to Question 70 appears on the next page

This is a sample form. Do not use for scanning.

Over the past month…

69f. How often was **cream cheese** (including low-fat) spread on your bagels or English muffins?

- ☐ Almost never or never (GO TO INTRODUCTION TO QUESTION 70)
- ☐ About ¼ of the time
- ☐ About ½ of the time
- ☐ About ¾ of the time
- ☐ Almost always or always

69g. Each time **cream cheese** was added to your bagels or English muffins, how much was usually added?

- ☐ Less than 1 tablespoon
- ☐ 1 to 2 tablespoons
- ☐ More than 2 tablespoons

The next questions ask about your intake of breads other than bagels or English muffins. First, we will ask about bread you ate as part of sandwiches only. Then we will ask about all other bread you ate.

70. How often did you eat **breads** or **rolls AS PART OF SANDWICHES** (including burger and hot dog rolls)?
(Please do not include fast food sandwiches.)

- ☐ NEVER (GO TO QUESTION 71)

- ☐ 1 time in past month
- ☐ 2–3 times in past month
- ☐ 1 time per week
- ☐ 2 times per week
- ☐ 3–4 times per week
- ☐ 5–6 times per week
- ☐ 1 time per day
- ☐ 2 or more times per day

70a. Each time you ate **breads** or **rolls AS PART OF SANDWICHES**, how many did you usually eat?

- ☐ 1 slice or ½ roll
- ☐ 2 slices or 1 roll
- ☐ More than 2 slices or more than 1 roll

70b. How often were the breads or rolls that you used for your sandwiches **white bread** (including burger and hot dog rolls)?

- ☐ Almost never or never
- ☐ About ¼ of the time
- ☐ About ½ of the time
- ☐ About ¾ of the time
- ☐ Almost always or always

Question 71 appears in the next column

70c. How often was **mayonnaise** or **mayonnaise-type dressing** (including low-fat) added to the breads or rolls used for your sandwiches?

- ☐ Almost never or never (GO TO QUESTION 70e)
- ☐ About ¼ of the time
- ☐ About ½ of the time
- ☐ About ¾ of the time
- ☐ Almost always or always

70d. Each time **mayonnaise** or **mayonnaise-type dressing** was added to the breads or rolls used for your sandwiches, how much was usually added?

- ☐ Less than 1 teaspoon
- ☐ 1 to 3 teaspoons
- ☐ More than 3 teaspoons

70e. How often was **margarine** (including low-fat) added to the breads or rolls used for your sandwiches?

- ☐ Almost never or never
- ☐ About ¼ of the time
- ☐ About ½ of the time
- ☐ About ¾ of the time
- ☐ Almost always or always

70f. How often was **butter** (including low-fat) added to the breads or rolls used for your sandwiches?

- ☐ Almost never or never
- ☐ About ¼ of the time
- ☐ About ½ of the time
- ☐ About ¾ of the time
- ☐ Almost always or always

70g. Each time **margarine** or **butter** was added to the breads or rolls used for your sandwiches, how much was usually added?

- ☐ Never added
- ☐ Less than 1 teaspoon
- ☐ 1 to 2 teaspoons
- ☐ More than 2 teaspoons

71. How often did you eat **breads** or **dinner rolls**, **NOT AS PART OF SANDWICHES**?

- ☐ NEVER (GO TO QUESTION 72)

- ☐ 1 time in past month
- ☐ 2–3 times in past month
- ☐ 1 time per week
- ☐ 2 times per week
- ☐ 3–4 times per week
- ☐ 5–6 times per week
- ☐ 1 time per day
- ☐ 2 or more times per day

Question 72 appears on the next page

This is a sample form. Do not use for scanning.

Over the past month…

71a. Each time you ate **breads** or **dinner rolls, NOT AS PART OF SANDWICHES**, how much did you usually eat?

- ☐ 1 slice or 1 dinner roll
- ☐ 2 slices or 2 dinner rolls
- ☐ More than 2 slices or 2 dinner rolls

71b. How often were the breads or rolls you ate **white bread**?

- ☐ Almost never or never
- ☐ About ¼ of the time
- ☐ About ½ of the time
- ☐ About ¾ of the time
- ☐ Almost always or always

71c. How often was **margarine** (including low-fat) added to your breads or rolls?

- ☐ Almost never or never
- ☐ About ¼ of the time
- ☐ About ½ of the time
- ☐ About ¾ of the time
- ☐ Almost always or always

71d. How often was **butter** (including low-fat) added to your breads or rolls?

- ☐ Almost never or never
- ☐ About ¼ of the time
- ☐ About ½ of the time
- ☐ About ¾ of the time
- ☐ Almost always or always

71e. Each time **margarine** or **butter** was added to your breads or rolls, how much was usually added?

- ☐ Never added
- ☐ Less than 1 teaspoon
- ☐ 1 to 2 teaspoons
- ☐ More than 2 teaspoons

71f. How often was **cream cheese** (including low-fat) added to your breads or rolls?

- ☐ Almost never or never (GO TO QUESTION 72)
- ☐ About ¼ of the time
- ☐ About ½ of the time
- ☐ About ¾ of the time
- ☐ Almost always or always

Question 72 appears in the next column

71g. Each time **cream cheese** was added to your breads or rolls, how much was usually added?

- ☐ Less than 1 tablespoon
- ☐ 1 to 2 tablespoons
- ☐ More than 2 tablespoons

72. How often did you eat **jam, jelly,** or **honey** on bagels, muffins, bread, rolls, or crackers?

- ☐ NEVER (GO TO QUESTION 73)
- ☐ 1 time in past month
- ☐ 2–3 times in past month
- ☐ 1 time per week
- ☐ 2 times per week
- ☐ 3–4 times per week
- ☐ 5–6 times per week
- ☐ 1 time per day
- ☐ 2 or more times per day

72a. Each time you ate **jam, jelly,** or **honey**, how much did you usually eat?

- ☐ Less than 1 teaspoon
- ☐ 1 to 3 teaspoons
- ☐ More than 3 teaspoons

73. How often did you eat **peanut butter** or **other nut butter**?

- ☐ NEVER (GO TO QUESTION 74)
- ☐ 1 time in past month
- ☐ 2–3 times in past month
- ☐ 1 time per week
- ☐ 2 times per week
- ☐ 3–4 times per week
- ☐ 5–6 times per week
- ☐ 1 time per day
- ☐ 2 or more times per day

73a. Each time you ate **peanut butter** or **other nut butter**, how much did you usually eat?

- ☐ Less than 1 tablespoon
- ☐ 1 to 2 tablespoons
- ☐ More than 2 tablespoons

74. How often did you eat **roast beef** or **steak IN SANDWICHES**?

- ☐ NEVER (GO TO QUESTION 75)
- ☐ 1 time in past month
- ☐ 2–3 times in past month
- ☐ 1 time per week
- ☐ 2 times per week
- ☐ 3–4 times per week
- ☐ 5–6 times per week
- ☐ 1 time per day
- ☐ 2 or more times per day

74a. Each time you ate **roast beef** or **steak IN SANDWICHES**, how much did you usually eat?

- ☐ Less than 1 slice or less than 2 ounces
- ☐ 1 to 2 slices or 2 to 4 ounces
- ☐ More than 2 slices or more than 4 ounces

Question 75 appears on the next page

This is a sample form. Do not use for scanning.

Over the past month…

75. How often did you eat **turkey** or **chicken COLD CUTS** (such as loaf, luncheon meat, turkey ham, turkey salami, or turkey pastrami)? (*We will ask about other turkey or chicken later.)*

- ☐ NEVER (GO TO QUESTION 76)
- ☐ 1 time in past month
- ☐ 2–3 times in past month
- ☐ 1 time per week
- ☐ 2 times per week
- ☐ 3–4 times per week
- ☐ 5–6 times per week
- ☐ 1 time per day
- ☐ 2 or more times per day

75a. Each time you ate **turkey or chicken COLD CUTS**, how much did you usually eat?

- ☐ Less than 1 slice
- ☐ 1 to 3 slices
- ☐ More than 3 slices

76. How often did you eat **luncheon** or **deli-style ham**? *(We will ask about other ham later.)*

- ☐ NEVER (GO TO QUESTION 77)
- ☐ 1 time in past month
- ☐ 2–3 times in past month
- ☐ 1 time per week
- ☐ 2 times per week
- ☐ 3–4 times per week
- ☐ 5–6 times per week
- ☐ 1 time per day
- ☐ 2 or more times per day

76a. Each time you ate **luncheon** or **deli-style ham**, how much did you usually eat?

- ☐ Less than 1 slice
- ☐ 1 to 3 slices
- ☐ More than 3 slices

76b. How often was the luncheon or deli-style ham you ate **light**, **low-fat**, or **fat-free**?

- ☐ Almost never or never
- ☐ About ¼ of the time
- ☐ About ½ of the time
- ☐ About ¾ of the time
- ☐ Almost always or always

77. How often did you eat **other cold cuts** or **luncheon meats** (such as bologna, salami, corned beef, pastrami, or others, including low-fat)? *(Please do not include ham, turkey, or chicken cold cuts.)*

- ☐ NEVER (GO TO QUESTION 78)
- ☐ 1 time in past month
- ☐ 2–3 times in past month
- ☐ 1 time per week
- ☐ 2 times per week
- ☐ 3–4 times per week
- ☐ 5–6 times per week
- ☐ 1 time per day
- ☐ 2 or more times per day

Question 78 appears in the next column

77a. Each time you ate **other cold cuts** or **luncheon meats**, how much did you usually eat?

- ☐ Less than 1 slice
- ☐ 1 to 3 slices
- ☐ More than 3 slices

77b. How often were the other cold cuts or luncheon meats you ate **light, low-fat**, or **fat-free**? *(Please do not include ham, turkey, or chicken cold cuts.)*

- ☐ Almost never or never
- ☐ About ¼ of the time
- ☐ About ½ of the time
- ☐ About ¾ of the time
- ☐ Almost always or always

78. How often did you eat **canned tuna** (including in salads, sandwiches, or casseroles)?

- ☐ NEVER (GO TO QUESTION 79)
- ☐ 1 time in past month
- ☐ 2–3 times in past month
- ☐ 1 time per week
- ☐ 2 times per week
- ☐ 3–4 times per week
- ☐ 5–6 times per week
- ☐ 1 time per day
- ☐ 2 or more times per day

78a. Each time you ate **canned tuna**, how much did you usually eat?

- ☐ Less than ¼ cup or less than 2 ounces
- ☐ ¼ to ½ cup or 2 to 3 ounces
- ☐ More than ½ cup or more than 3 ounces

78b. How often was the canned tuna you ate **water-packed**?

- ☐ Almost never or never
- ☐ About ¼ of the time
- ☐ About ½ of the time
- ☐ About ¾ of the time
- ☐ Almost always or always

78c. How often was the canned tuna you ate **prepared with mayonnaise** or **other dressing** (including low-fat)?

- ☐ Almost never or never
- ☐ About ¼ of the time
- ☐ About ½ of the time
- ☐ About ¾ of the time
- ☐ Almost always or always

Question 79 appears on the next page

This is a sample form. Do not use for scanning.

Over the past month…

79. How often did you eat **GROUND chicken** or **turkey**? *(We will ask about other chicken and turkey later.)*

- ☐ NEVER (GO TO QUESTION 80)
- ☐ 1 time in past month
- ☐ 2–3 times in past month
- ☐ 1 time per week
- ☐ 2 times per week
- ☐ 3–4 times per week
- ☐ 5–6 times per week
- ☐ 1 time per day
- ☐ 2 or more times per day

79a. Each time you ate **GROUND chicken** or **turkey**, how much did you usually eat?

- ☐ Less than 2 ounces or less than ½ cup
- ☐ 2 to 4 ounces or ½ to 1 cup
- ☐ More than 4 ounces or more than 1 cup

80. How often did you eat **beef hamburgers** or **cheeseburgers** from a **FAST FOOD** or **OTHER RESTAURANT**?

- ☐ NEVER (GO TO QUESTION 81)
- ☐ 1 time in past month
- ☐ 2–3 times in past month
- ☐ 1 time per week
- ☐ 2 times per week
- ☐ 3–4 times per week
- ☐ 5–6 times per week
- ☐ 1 time per day
- ☐ 2 or more times per day

80a. Each time you ate **beef hamburgers** or **cheeseburgers** from a **FAST FOOD** or **OTHER RESTAURANT**, what size did you usually eat?

- ☐ Small hamburger (such as a regular Burger King or McDonald's Hamburger)
- ☐ Medium (such as McDonald's or Burger King Double Burger or Cheeseburger)
- ☐ Large (such as Burger King Whopper or Double Whopper or a McDonald's Double Quarter Pounder)

80b. Each time you ate **beef hamburgers** or **cheeseburgers** from a **FAST FOOD** or **OTHER RESTAURANT**, how much did you usually eat?

- ☐ Less than 1 burger
- ☐ 1 burger
- ☐ More than 1 burger

80c. How often did you have **cheeseburgers** rather than **hamburgers**?

- ☐ Almost never or never
- ☐ About ¼ of the time
- ☐ About ½ of the time
- ☐ About ¾ of the time
- ☐ Almost always or always

Question 81 appears in the next column

81. How often did you eat **beef hamburgers** or **cheeseburgers** that were **NOT FROM A FAST FOOD** or **OTHER RESTAURANT**?

- ☐ NEVER (GO TO QUESTION 82)
- ☐ 1 time in past month
- ☐ 2–3 times in past month
- ☐ 1 time per week
- ☐ 2 times per week
- ☐ 3–4 times per week
- ☐ 5–6 times per week
- ☐ 1 time per day
- ☐ 2 or more times per day

81a. Each time you ate **beef hamburgers** or **cheeseburgers** that were **NOT FROM A FAST FOOD** or **OTHER RESTAURANT**, how much did you usually eat?

- ☐ Less than 1 patty or less than 2 ounces
- ☐ 1 patty or 2 to 4 ounces
- ☐ More than 1 patty or more than 4 ounces

81b. How often were these beef hamburgers or cheeseburgers made with **lean ground beef**?

- ☐ Almost never or never
- ☐ About ¼ of the time
- ☐ About ½ of the time
- ☐ About ¾ of the time
- ☐ Almost always or always

82. How often did you eat **ground beef in mixtures** (such as meatballs, casseroles, chili, or meatloaf)?

- ☐ NEVER (GO TO QUESTION 83)
- ☐ 1 time in past month
- ☐ 2–3 times in past month
- ☐ 1 time per week
- ☐ 2 times per week
- ☐ 3–4 times per week
- ☐ 5–6 times per week
- ☐ 1 time per day
- ☐ 2 or more times per day

82a. Each time you ate **ground beef in mixtures**, how much did you usually eat?

- ☐ Less than 3 ounces or less than ½ cup
- ☐ 3 to 8 ounces or ½ to 1 cup
- ☐ More than 8 ounces or more than 1 cup

83. How often did you eat **hot dogs** or **frankfurters**? *(Please do not include sausages or vegetarian hot dogs.)*

- ☐ NEVER (GO TO QUESTION 84)
- ☐ 1 time in past month
- ☐ 2–3 times in past month
- ☐ 1 time per week
- ☐ 2 times per week
- ☐ 3–4 times per week
- ☐ 5–6 times per week
- ☐ 1 time per day
- ☐ 2 or more times per day

Question 84 appears on the next page

This is a sample form. Do not use for scanning.

Over the past month…

83a. Each time you ate **hot dogs** or **frankfurters**, how many did you usually eat?

- ☐ Less than 1 hot dog
- ☐ 1 to 2 hot dogs
- ☐ More than 2 hot dogs

83b. How often were the hot dogs or frankfurters you ate **light** or **low-fat**?

- ☐ Almost never or never
- ☐ About ¼ of the time
- ☐ About ½ of the time
- ☐ About ¾ of the time
- ☐ Almost always or always

84. How often did you eat **beef mixtures** (such as beef stew, beef pot pie, beef and noodles, or beef and vegetables)?

- ☐ NEVER (GO TO QUESTION 85)

- ☐ 1 time in past month
- ☐ 2–3 times in past month
- ☐ 1 time per week
- ☐ 2 times per week
- ☐ 3–4 times per week
- ☐ 5–6 times per week
- ☐ 1 time per day
- ☐ 2 or more times per day

84a. Each time you ate **beef mixtures**, how much did you usually eat?

- ☐ Less than 1 cup
- ☐ 1 to 2 cups
- ☐ More than 2 cups

85. How often did you eat **roast beef** or **pot roast**? *(Please do not include roast beef or pot roast in sandwiches.)*

- ☐ NEVER (GO TO QUESTION 86)

- ☐ 1 time in past month
- ☐ 2–3 times in past month
- ☐ 1 time per week
- ☐ 2 times per week
- ☐ 3–4 times per week
- ☐ 5–6 times per week
- ☐ 1 time per day
- ☐ 2 or more times per day

85a. Each time you ate **roast beef** or **pot roast**, how much did you usually eat?

- ☐ Less than 2 ounces
- ☐ 2 to 5 ounces
- ☐ More than 5 ounces

Question 86 appears in the next column

86. How often did you eat **steak** (beef)? (*Please do not include steak in sandwiches*)

- ☐ NEVER (GO TO QUESTION 87)

- ☐ 1 time in past month
- ☐ 2–3 times in past month
- ☐ 1 time per week
- ☐ 2 times per week
- ☐ 3–4 times per week
- ☐ 5–6 times per week
- ☐ 1 time per day
- ☐ 2 or more times per day

86a. Each time you ate **steak** (beef), how much did you usually eat?

- ☐ Less than 3 ounces
- ☐ 3 to 7 ounces
- ☐ More than 7 ounces

86b. How often was the steak you ate **lean steak**?

- ☐ Almost never or never
- ☐ About ¼ of the time
- ☐ About ½ of the time
- ☐ About ¾ of the time
- ☐ Almost always or always

87. How often did you eat **pork** or **beef spareribs**?

- ☐ NEVER (GO TO QUESTION 88)

- ☐ 1 time in past month
- ☐ 2–3 times in past month
- ☐ 1 time per week
- ☐ 2 times per week
- ☐ 3–4 times per week
- ☐ 5–6 times per week
- ☐ 1 time per day
- ☐ 2 or more times per day

87a. Each time you ate **pork** or **beef spareribs**, how much did you usually eat?

- ☐ Less than 4 ribs
- ☐ 4 to 12 ribs
- ☐ More than 12 ribs

88. How often did you eat **roast turkey, turkey cutlets,** or **turkey nuggets** (including in sandwiches)?

- ☐ NEVER (GO TO QUESTION 89)

- ☐ 1 time in past month
- ☐ 2–3 times in past month
- ☐ 1 time per week
- ☐ 2 times per week
- ☐ 3–4 times per week
- ☐ 5–6 times per week
- ☐ 1 time per day
- ☐ 2 or more times per day

88a. Each time you ate **roast turkey, turkey cutlets,** or **turkey nuggets**, how much did you usually eat? *(Please note: 4 to 8 turkey nuggets = 3 ounces.)*

- ☐ Less than 2 ounces
- ☐ 2 to 4 ounces
- ☐ More than 4 ounces

Question 89 appears on the next page

This is a sample form. Do not use for scanning.

Over the <u>past month</u>…

89. How often did you eat **chicken mixtures** (such as salads, sandwiches, casseroles, stews, or other mixtures)?

- ☐ NEVER (GO TO QUESTION 90)

- ☐ 1 time in past month
- ☐ 2–3 times in past month
- ☐ 1 time per week
- ☐ 2 times per week
- ☐ 3–4 times per week
- ☐ 5–6 times per week
- ☐ 1 time per day
- ☐ 2 or more times per day

89a. Each time you ate **chicken mixtures**, how much did you usually eat?

- ☐ Less than ½ cup
- ☐ ½ to 1½ cups
- ☐ More than 1½ cups

90. How often did you eat **baked, broiled, roasted, stewed,** or **fried chicken** (including nuggets)? *(Please do not include chicken in mixtures.)*

- ☐ NEVER (GO TO QUESTION 91)

- ☐ 1 time in past month
- ☐ 2–3 times in past month
- ☐ 1 time per week
- ☐ 2 times per week
- ☐ 3–4 times per week
- ☐ 5–6 times per week
- ☐ 1 time per day
- ☐ 2 or more times per day

90a. Each time you ate **baked, broiled, roasted, stewed,** or **fried chicken** (including nuggets), how much did you usually eat?

- ☐ Less than 2 drumsticks or wings, less than 1 breast or thigh, or less than 4 nuggets
- ☐ 2 drumsticks or wings, 1 breast or thigh, or 4 to 8 nuggets
- ☐ More than 2 drumsticks or wings, more than 1 breast or thigh, or more than 8 nuggets

90b. How often was the chicken you ate **fried chicken** (including deep fried) or **chicken nuggets**?

- ☐ Almost never or never
- ☐ About ¼ of the time
- ☐ About ½ of the time
- ☐ About ¾ of the time
- ☐ Almost always or always

90c. How often was the chicken you ate **WHITE meat**?

- ☐ Almost never or never
- ☐ About ¼ of the time
- ☐ About ½ of the time
- ☐ About ¾ of the time
- ☐ Almost always or always

Question 91 appears in the next column

90d. How often did you eat chicken **WITH skin**?

- ☐ Almost never or never
- ☐ About ¼ of the time
- ☐ About ½ of the time
- ☐ About ¾ of the time
- ☐ Almost always or always

91. How often did you eat **baked ham** or **ham steak**?

- ☐ NEVER (GO TO QUESTION 92)

- ☐ 1 time in past month
- ☐ 2–3 times in past month
- ☐ 1 time per week
- ☐ 2 times per week
- ☐ 3–4 times per week
- ☐ 5–6 times per week
- ☐ 1 time per day
- ☐ 2 or more times per day

91a. Each time you ate **baked ham** or **ham steak**, how much did you usually eat?

- ☐ Less than 1 ounce
- ☐ 1 to 3 ounces
- ☐ More than 3 ounces

92. How often did you eat **pork** (including chops, roasts, and in mixed dishes)? *(Please do not include ham, ham steak, or sausage.)*

- ☐ NEVER (GO TO QUESTION 93)

- ☐ 1 time in past month
- ☐ 2–3 times in past month
- ☐ 1 time per week
- ☐ 2 times per week
- ☐ 3–4 times per week
- ☐ 5–6 times per week
- ☐ 1 time per day
- ☐ 2 or more times per day

92a. Each time you ate **pork**, how much did you usually eat?

- ☐ Less than 2 ounces or less than 1 chop
- ☐ 2 to 5 ounces or 1 chop
- ☐ More than 5 ounces or more than 1 chop

93. How often did you eat **gravy** on meat, chicken, potatoes, rice, etc.?

- ☐ NEVER (GO TO QUESTION 94)

- ☐ 1 time in past month
- ☐ 2–3 times in past month
- ☐ 1 time per week
- ☐ 2 times per week
- ☐ 3–4 times per week
- ☐ 5–6 times per week
- ☐ 1 time per day
- ☐ 2 or more times per day

93a. Each time you ate **gravy** on meat, chicken, potatoes, rice, etc., how much did you usually eat?

- ☐ Less than ⅛ cup
- ☐ ⅛ to ½ cup
- ☐ More than ½ cup

Question 94 appears on the next page

This is a sample form. Do not use for scanning.

Over the past month…

94. How often did you eat **liver** (all kinds) or **liverwurst**?

- ☐ NEVER (GO TO QUESTION 95)
- ☐ 1 time in past month
- ☐ 2–3 times in past month
- ☐ 1 time per week
- ☐ 2 times per week
- ☐ 3–4 times per week
- ☐ 5–6 times per week
- ☐ 1 time per day
- ☐ 2 or more times per day

94a. Each time you ate **liver** or **liverwurst**, how much did you usually eat?

- ☐ Less than 1 ounce
- ☐ 1 to 4 ounces
- ☐ More than 4 ounces

95. How often did you eat **bacon** (including low-fat)?

- ☐ NEVER (GO TO QUESTION 96)
- ☐ 1 time in past month
- ☐ 2–3 times in past month
- ☐ 1 time per week
- ☐ 2 times per week
- ☐ 3–4 times per week
- ☐ 5–6 times per week
- ☐ 1 time per day
- ☐ 2 or more times per day

95a. Each time you ate **bacon**, how much did you usually eat?

- ☐ Fewer than 2 slices
- ☐ 2 to 3 slices
- ☐ More than 3 slices

95b. How often was the bacon you ate **light, low-fat,** or **lean**?

- ☐ Almost never or never
- ☐ About ¼ of the time
- ☐ About ½ of the time
- ☐ About ¾ of the time
- ☐ Almost always or always

96. How often did you eat **sausage** (including low-fat)?

- ☐ NEVER (GO TO QUESTION 97)
- ☐ 1 time in past month
- ☐ 2–3 times in past month
- ☐ 1 time per week
- ☐ 2 times per week
- ☐ 3–4 times per week
- ☐ 5–6 times per week
- ☐ 1 time per day
- ☐ 2 or more times per day

96a. Each time you ate **sausage**, how much did you usually eat?

- ☐ Less than 1 patty or 2 links
- ☐ 1 to 3 patties or 2 to 5 links
- ☐ More than 3 patties or 5 links

Question 97 appears in the next column

96b. How often was the sausage you ate **light, low-fat**, or **lean**?

- ☐ Almost never or never
- ☐ About ¼ of the time
- ☐ About ½ of the time
- ☐ About ¾ of the time
- ☐ Almost always or always

97. How often did you eat **fried shellfish** (such as crab, lobster, shrimp)?

- ☐ NEVER (GO TO QUESTION 98)
- ☐ 1 time in past month
- ☐ 2–3 times in past month
- ☐ 1 time per week
- ☐ 2 times per week
- ☐ 3–4 times per week
- ☐ 5–6 times per week
- ☐ 1 time per day
- ☐ 2 or more times per day

97a. Each time you ate **fried shellfish**, how much did you usually eat?

- ☐ Less than 2 ounces
- ☐ 2 to 4 ounces
- ☐ More than 4 ounces

98. How often did you eat **shellfish** (such as crab, lobster, shrimp) **that was NOT FRIED**?

- ☐ NEVER (GO TO QUESTION 99)
- ☐ 1 time in past month
- ☐ 2–3 times in past month
- ☐ 1 time per week
- ☐ 2 times per week
- ☐ 3–4 times per week
- ☐ 5–6 times per week
- ☐ 1 time per day
- ☐ 2 or more times per day

98a. Each time you ate **shellfish that was NOT FRIED**, how much did you usually eat?

- ☐ Less than 1 ounce
- ☐ 1 to 4 ounces
- ☐ More than 4 ounces

99. How often did you eat **salmon, fresh tuna or trout**?

- ☐ NEVER (GO TO QUESTION 100)
- ☐ 1 time in past month
- ☐ 2–3 times in past month
- ☐ 1 time per week
- ☐ 2 times per week
- ☐ 3–4 times per week
- ☐ 5–6 times per week
- ☐ 1 time per day
- ☐ 2 or more times per day

99a. Each time you ate **salmon, fresh tuna or trout**, how much did you usually eat?

- ☐ Less than 2 ounces
- ☐ 2 to 6 ounces
- ☐ More than 6 ounces

Question 100 appears on the next page

This is a sample form. Do not use for scanning.

Over the past month…

100. How often did you eat **fish sticks** or other **fried fish** (not including shellfish)?

- ☐ NEVER (GO TO QUESTION 101)
- ☐ 1 time in past month
- ☐ 2–3 times in past month
- ☐ 1 time per week
- ☐ 2 times per week
- ☐ 3–4 times per week
- ☐ 5–6 times per week
- ☐ 1 time per day
- ☐ 2 or more times per day

100a. Each time you ate **fish sticks** or **other fried fish**, how much did you usually eat?

- ☐ Less than 2 ounces or less than 1 fillet
- ☐ 2 to 7 ounces or 1 fillet
- ☐ More than 7 ounces or more than 1 fillet

101. How often did you eat **other fish that was NOT FRIED** (not including shellfish)?

- ☐ NEVER (GO TO INTRODUCTION TO QUESTION 102)
- ☐ 1 time in past month
- ☐ 2–3 times in past month
- ☐ 1 time per week
- ☐ 2 times per week
- ☐ 3–4 times per week
- ☐ 5–6 times per week
- ☐ 1 time per day
- ☐ 2 or more times per day

101a. Each time you ate **other fish that was NOT FRIED**, how much did you usually eat?

- ☐ Less than 2 ounces or less than 1 fillet
- ☐ 2 to 5 ounces or 1 fillet
- ☐ More than 5 ounces or more than 1 fillet

Now think about all the meat, poultry, and fish you ate in the past month and how they were prepared.

102. How often was **oil, butter, margarine**, **or other fat** used to **FRY, SAUTE, BASTE, OR MARINATE** any meat, poultry, or fish you ate? *(Please do not include deep frying.)*

- ☐ NEVER (GO TO QUESTION 103)
- ☐ 1 time in past month
- ☐ 2–3 times in past month
- ☐ 1 time per week
- ☐ 2 times per week
- ☐ 3–4 times per week
- ☐ 5–6 times per week
- ☐ 1 time per day
- ☐ 2 or more times per day

Question 103 appears in the next column

102a. Which of the following **fats** were regularly used to prepare your meat, poultry, or fish? ***(Mark all that apply.)***

- ☐ Margarine (including low-fat)
- ☐ Butter (including low-fat)
- ☐ Lard, fatback, or bacon fat
- ☐ Olive oil
- ☐ Corn oil
- ☐ Canola or rapeseed oil
- ☐ Oil spray (such as Pam or others)
- ☐ Other kinds of oils
- ☐ None of the above

103. How often did you eat **tofu, soy burgers,** or **soy meat-substitutes**?

- ☐ NEVER (GO TO QUESTION 104)
- ☐ 1 time in past month
- ☐ 2–3 times in past month
- ☐ 1 time per week
- ☐ 2 times per week
- ☐ 3–4 times per week
- ☐ 5–6 times per week
- ☐ 1 time per day
- ☐ 2 or more times per day

103a. Each time you ate **tofu, soy burgers,** or **soy meat-substitutes**, how much did you usually eat?

- ☐ Less than ¼ cup or less than 2 ounces
- ☐ ¼ to ½ cup or 2 to 4 ounces
- ☐ More than ½ cup or more than 4 ounces

104. How often did you eat **soups**?

- ☐ NEVER (GO TO QUESTION 105)
- ☐ 1 time in past month
- ☐ 2–3 times in past month
- ☐ 1 time per week
- ☐ 2 times per week
- ☐ 3–4 times per week
- ☐ 5–6 times per week
- ☐ 1 time per day
- ☐ 2 or more times per day

104a. Each time you ate **soup**, how much did you usually eat?

- ☐ Less than 1 cup
- ☐ 1 to 2 cups
- ☐ More than 2 cups

104b. How often were the soups you ate **bean soups**?

- ☐ Almost never or never
- ☐ About ¼ of the time
- ☐ About ½ of the time
- ☐ About ¾ of the time
- ☐ Almost always or always

Question 105 appears on the next page

This is a sample form. Do not use for scanning.

Over the past month…

104c. How often were the soups you ate **cream soups** (including chowders)?

- ☐ Almost never or never
- ☐ About ¼ of the time
- ☐ About ½ of the time
- ☐ About ¾ of the time
- ☐ Almost always or always

104d. How often were the soups you ate **tomato** or **vegetable soups**?

- ☐ Almost never or never
- ☐ About ¼ of the time
- ☐ About ½ of the time
- ☐ About ¾ of the time
- ☐ Almost always or always

104e. How often were the soups you ate **broth soups** (including chicken) **with** or **without noodles** or **rice**?

- ☐ Almost never or never
- ☐ About ¼ of the time
- ☐ About ½ of the time
- ☐ About ¾ of the time
- ☐ Almost always or always

105. How often did you eat **pizza**?

- ☐ NEVER (GO TO QUESTION 106)
- ☐ 1 time in past month
- ☐ 2–3 times in past month
- ☐ 1 time per week
- ☐ 2 times per week
- ☐ 3–4 times per week
- ☐ 5–6 times per week
- ☐ 1 time per day
- ☐ 2 or more times per day

105a. Each time you ate **pizza**, how much did you usually eat?

- ☐ Less than 1 slice or less than 1 mini pizza
- ☐ 1 to 3 slices or 1 mini pizza
- ☐ More than 3 slices or more than 1 mini pizza

105b. How often did you eat pizza with **pepperoni, sausage**, or **other meat**?

- ☐ Almost never or never
- ☐ About ¼ of the time
- ☐ About ½ of the time
- ☐ About ¾ of the time
- ☐ Almost always or always

Question 106 appears in the next column

106. How often did you eat **crackers**?

- ☐ NEVER (GO TO QUESTION 107)
- ☐ 1 time in past month
- ☐ 2–3 times in past month
- ☐ 1 time per week
- ☐ 2 times per week
- ☐ 3–4 times per week
- ☐ 5–6 times per week
- ☐ 1 time per day
- ☐ 2 or more times per day

106a. Each time you ate **crackers**, how many did you usually eat?

- ☐ Fewer than 4 crackers
- ☐ 4 to 10 crackers
- ☐ More than 10 crackers

107. How often did you eat **corn bread** or **corn muffins**?

- ☐ NEVER (GO TO QUESTION 108)
- ☐ 1 time in past month
- ☐ 2–3 times in past month
- ☐ 1 time per week
- ☐ 2 times per week
- ☐ 3–4 times per week
- ☐ 5–6 times per week
- ☐ 1 time per day
- ☐ 2 or more times per day

107a. Each time you ate **corn bread** or **corn muffins,** how much did you usually eat?

- ☐ Less than 1 piece or muffin
- ☐ 1 to 2 pieces or muffins
- ☐ More than 2 pieces or muffins

108. How often did you eat **biscuits**?

- ☐ NEVER (GO TO QUESTION 109)
- ☐ 1 time in past month
- ☐ 2–3 times in past month
- ☐ 1 time per week
- ☐ 2 times per week
- ☐ 3–4 times per week
- ☐ 5–6 times per week
- ☐ 1 time per day
- ☐ 2 or more times per day

108a. Each time you ate **biscuits**, how many did you usually eat?

- ☐ Fewer than 1 biscuit
- ☐ 1 to 2 biscuits
- ☐ More than 2 biscuits

109. How often did you eat **potato chips** (including low-fat, fat-free, or low-salt)?

- ☐ NEVER (GO TO QUESTION 110)
- ☐ 1 time in past month
- ☐ 2–3 times in past month
- ☐ 1 time per week
- ☐ 2 times per week
- ☐ 3–4 times per week
- ☐ 5–6 times per week
- ☐ 1 time per day
- ☐ 2 or more times per day

Question 110 appears on the next page

This is a sample form. Do not use for scanning.

Over the past month…

109a. Each time you ate **potato chips**, how much did you usually eat?

- ☐ Fewer than 10 chips or less than 1 cup
- ☐ 10 to 25 chips or 1 to 2 cups
- ☐ More than 25 chips or more than 2 cups

109b. How often were the potato chips you ate **fat-free**? *(Please do not include reduced-fat chips.)*

- ☐ Almost never or never
- ☐ About ¼ of the time
- ☐ About ½ of the time
- ☐ About ¾ of the time
- ☐ Almost always or always

110. How often did you eat **corn chips or tortilla chips** (including low-fat, fat-free, or low-salt)?

- ☐ NEVER (GO TO QUESTION 111)

- ☐ 1 time in past month
- ☐ 2–3 times in past month
- ☐ 1 time per week
- ☐ 2 times per week
- ☐ 3–4 times per week
- ☐ 5–6 times per week
- ☐ 1 time per day
- ☐ 2 or more times per day

110a. Each time you ate **corn chips**, how much did you usually eat?

- ☐ Fewer than 10 chips or less than 1 cup
- ☐ 10 to 25 chips or 1 to 1½ cups
- ☐ More than 25 chips or more than 1½ cups

110b. How often were the corn chips or tortilla chips you ate **fat-free**? *(Please do not include reduced-fat chips.)*

- ☐ Almost never or never
- ☐ About ¼ of the time
- ☐ About ½ of the time
- ☐ About ¾ of the time
- ☐ Almost always or always

111. How often did you eat **popcorn** (including low-fat)?

- ☐ NEVER (GO TO QUESTION 112)

- ☐ 1 time in past month
- ☐ 2–3 times in past month
- ☐ 1 time per week
- ☐ 2 times per week
- ☐ 3–4 times per week
- ☐ 5–6 times per week
- ☐ 1 time per day
- ☐ 2 or more times per day

Question 112 appears in the next column

111a. Each time you ate **popcorn**, how much did you usually eat?

- ☐ Less than 2 cups, popped
- ☐ 2 to 5 cups, popped
- ☐ More than 5 cups, popped

112. How often did you eat **pretzels**?

- ☐ NEVER (GO TO QUESTION 113)

- ☐ 1 time in past month
- ☐ 2–3 times in past month
- ☐ 1 time per week
- ☐ 2 times per week
- ☐ 3–4 times per week
- ☐ 5–6 times per week
- ☐ 1 time per day
- ☐ 2 or more times per day

112a. Each time you ate **pretzels**, how many did you usually eat?

- ☐ Fewer than 5 average twists
- ☐ 5 to 20 average twists
- ☐ More than 20 average twists

113. How often did you eat **peanuts, walnuts, seeds,** or **other nuts**?

- ☐ NEVER (GO TO QUESTION 114)

- ☐ 1 time in past month
- ☐ 2–3 times in past month
- ☐ 1 time per week
- ☐ 2 times per week
- ☐ 3–4 times per week
- ☐ 5–6 times per week
- ☐ 1 time per day
- ☐ 2 or more times per day

113a. Each time you ate **peanuts, walnuts, seeds,** or **other nuts**, how much did you usually eat?

- ☐ Less than ¼ cup
- ☐ ¼ to ½ cup
- ☐ More than ½ cup

114. How often did you eat **energy, high-protein**, or **breakfast bars** (such as Power Bars, Balance, Clif, or others)?

- ☐ NEVER (GO TO QUESTION 115)

- ☐ 1 time in past month
- ☐ 2–3 times in past month
- ☐ 1 time per week
- ☐ 2 times per week
- ☐ 3–4 times per week
- ☐ 5–6 times per week
- ☐ 1 time per day
- ☐ 2 or more times per day

114a. Each time you ate **energy, high-protein**, or **breakfast bars**, how much did you usually eat?

- ☐ Less than 1 bar
- ☐ 1 bar
- ☐ More than 1 bar

Question 115 appears on the next page

This is a sample form. Do not use for scanning.

Over the past month…

115. How often did you eat **yogurt** (NOT including frozen yogurt)?

- ☐ NEVER (GO TO QUESTION 116)
- ☐ 1 time in past month
- ☐ 2–3 times in past month
- ☐ 1 time per week
- ☐ 2 times per week
- ☐ 3–4 times per week
- ☐ 5–6 times per week
- ☐ 1 time per day
- ☐ 2 or more times per day

115a. Each time you ate **yogurt**, how much did you usually eat?

- ☐ Less than ½ cup or less than 1 container
- ☐ ½ to 1 cup or 1 container
- ☐ More than 1 cup or more than 1 container

115b. How often was the **yogurt** you ate **low-fat** or **fat-free**?

- ☐ Almost never or never
- ☐ About ¼ of the time
- ☐ About ½ of the time
- ☐ About ¾ of the time
- ☐ Almost always or always

116. How often did you eat **cottage cheese** (including low-fat)?

- ☐ NEVER (GO TO QUESTION 117)
- ☐ 1 time in past month
- ☐ 2–3 times in past month
- ☐ 1 time per week
- ☐ 2 times per week
- ☐ 3–4 times per week
- ☐ 5–6 times per week
- ☐ 1 time per day
- ☐ 2 or more times per day

116a. Each time you ate **cottage cheese**, how much did you usually eat?

- ☐ Less than ¼ cup
- ☐ ¼ to 1 cup
- ☐ More than 1 cup

117. How often did you eat **cheese** (including low-fat; including on cheeseburgers or in sandwiches or subs)?

- ☐ NEVER (GO TO QUESTION 118)
- ☐ 1 time in past month
- ☐ 2–3 times in past month
- ☐ 1 time per week
- ☐ 2 times per week
- ☐ 3–4 times per week
- ☐ 5–6 times per week
- ☐ 1 time per day
- ☐ 2 or more times per day

Question 118 appears in the next column

117a. Each time you ate **cheese**, how much did you usually eat?

- ☐ Less than ½ ounce or less than 1 slice
- ☐ ½ to 1½ ounces or 1 slice
- ☐ More than 1½ ounces or more than 1 slice

117b. How often was the cheese you ate **low-fat or fat-free**?

- ☐ Almost never or never
- ☐ About ¼ of the time
- ☐ About ½ of the time
- ☐ About ¾ of the time
- ☐ Almost always or always

118. How often did you eat **frozen yogurt, sorbet,** or **ices** (including low-fat or fat-free)?

- ☐ NEVER (GO TO QUESTION 119)
- ☐ 1 time in past month
- ☐ 2–3 times in past month
- ☐ 1 time per week
- ☐ 2 times per week
- ☐ 3–4 times per week
- ☐ 5–6 times per week
- ☐ 1 time per day
- ☐ 2 or more times per day

118a. Each time you ate **frozen yogurt, sorbet,** or **ices**, how much did you usually eat?

- ☐ Less than ½ cup or less than 1 scoop
- ☐ ½ to 1 cup or 1 to 2 scoops
- ☐ More than 1 cup or more than 2 scoops

119. How often did you eat **ice cream, ice cream bars,** or **sherbet** (including low-fat or fat-free)?

- ☐ NEVER (GO TO QUESTION 120)
- ☐ 1 time in past month
- ☐ 2–3 times in past month
- ☐ 1 time per week
- ☐ 2 times per week
- ☐ 3–4 times per week
- ☐ 5–6 times per week
- ☐ 1 time per day
- ☐ 2 or more times per day

119a. Each time you ate **ice cream, ice cream bars,** or **sherbet**, how much did you usually eat?

- ☐ Less than ½ cup or less than 1 scoop
- ☐ ½ to 1½ cups or 1 to 2 scoops
- ☐ More than 1½ cups or more than 2 scoops

119b. How often was the ice cream you ate **light, low-fat,** or **fat-free ice cream** or **sherbet**?

- ☐ Almost never or never
- ☐ About ¼ of the time
- ☐ About ½ of the time
- ☐ About ¾ of the time
- ☐ Almost always or always

Question 120 appears on the next page

This is a sample form. Do not use for scanning.

Over the past month…

120. How often did you eat **cake** (including low-fat or fat-free)?

- ☐ NEVER (GO TO QUESTION 121)
- ☐ 1 time in past month
- ☐ 2–3 times in past month
- ☐ 1 time per week
- ☐ 2 times per week
- ☐ 3–4 times per week
- ☐ 5–6 times per week
- ☐ 1 time per day
- ☐ 2 or more times per day

120a. Each time you ate **cake**, how much did you usually eat?

- ☐ Less than 1 medium piece
- ☐ 1 medium piece
- ☐ More than 1 medium piece

121. How often did you eat **cookies** or **brownies** (including low-fat or fat-free)?

- ☐ NEVER (GO TO QUESTION 122)
- ☐ 1 time in past month
- ☐ 2–3 times in past month
- ☐ 1 time per week
- ☐ 2 times per week
- ☐ 3–4 times per week
- ☐ 5–6 times per week
- ☐ 1 time per day
- ☐ 2 or more times per day

121a. Each time you ate **cookies** or **brownies**, how much did you usually eat?

- ☐ Less than 2 cookies or 1 small brownie
- ☐ 2 to 4 cookies or 1 medium brownie
- ☐ More than 4 cookies or 1 large brownie

122. How often did you eat **doughnuts**, **sweet rolls**, **Danish**, or **pop-tarts**?

- ☐ NEVER (GO TO QUESTION 123)
- ☐ 1 time in past month
- ☐ 2–3 times in past month
- ☐ 1 time per week
- ☐ 2 times per week
- ☐ 3–4 times per week
- ☐ 5–6 times per week
- ☐ 1 time per day
- ☐ 2 or more times per day

122a. Each time you ate **doughnuts, sweet rolls, Danish,** or **pop-tarts**, how much did you usually eat?

- ☐ Less than 1 piece
- ☐ 1 to 2 pieces
- ☐ More than 2 pieces

Question 123 appears in the next column

123. How often did you eat **sweet muffins** or **dessert breads** (including low-fat or fat-free)?

- ☐ NEVER (GO TO QUESTION 124)
- ☐ 1 time in past month
- ☐ 2–3 times in past month
- ☐ 1 time per week
- ☐ 2 times per week
- ☐ 3–4 times per week
- ☐ 5–6 times per week
- ☐ 1 time per day
- ☐ 2 or more times per day

123a. Each time you ate **sweet muffins or dessert breads**, how much did you usually eat?

- ☐ Less than 1 medium piece
- ☐ 1 medium piece
- ☐ More than 1 medium piece

124. How often did you eat **fruit crisp, cobbler,** or **strudel**?

- ☐ NEVER (GO TO QUESTION 125)
- ☐ 1 time in past month
- ☐ 2–3 times in past month
- ☐ 1 time per week
- ☐ 2 times per week
- ☐ 3–4 times per week
- ☐ 5–6 times per week
- ☐ 1 time per day
- ☐ 2 or more times per day

124a. Each time you ate **fruit crisp, cobbler,** or **strudel,** how much did you usually eat?

- ☐ Less than ½ cup
- ☐ ½ to 1 cup
- ☐ More than 1 cup

125. How often did you eat **pie**?

- ☐ NEVER (GO TO QUESTION 126)
- ☐ 1 time in past month
- ☐ 2–3 times in past month
- ☐ 1 time per week
- ☐ 2 times per week
- ☐ 3–4 times per week
- ☐ 5–6 times per week
- ☐ 1 time per day
- ☐ 2 or more times per day

125a. Each time you ate **pie**, how much did you usually eat?

- ☐ Less than ⅛ of a pie
- ☐ About ⅛ of a pie
- ☐ More than ⅛ of a pie

Question 126 appears on the next page

This is a sample form. Do not use for scanning.

Over the <u>past month</u>…

The next four questions ask about the kinds of pie you ate. Please read all four questions before answering.

125b. How often were the pies you ate **fruit pie** (such as apple, blueberry, others)?

- ☐ Almost never or never
- ☐ About ¼ of the time
- ☐ About ½ of the time
- ☐ About ¾ of the time
- ☐ Almost always or always

125c. How often were the pies you ate **cream, pudding, custard,** or **meringue pie**?

- ☐ Almost never or never
- ☐ About ¼ of the time
- ☐ About ½ of the time
- ☐ About ¾ of the time
- ☐ Almost always or always

125d. How often were the pies you ate **pumpkin** or **sweet potato pie**?

- ☐ Almost never or never
- ☐ About ¼ of the time
- ☐ About ½ of the time
- ☐ About ¾ of the time
- ☐ Almost always or always

125e. How often were the pies you ate **pecan pie**?

- ☐ Almost never or never
- ☐ About ¼ of the time
- ☐ About ½ of the time
- ☐ About ¾ of the time
- ☐ Almost always or always

126. How often did you eat **chocolate candy**?

- ☐ NEVER (GO TO QUESTION 127)
- ☐ 1 time in past month
- ☐ 2–3 times in past month
- ☐ 1 time per week
- ☐ 2 times per week
- ☐ 3–4 times per week
- ☐ 5–6 times per week
- ☐ 1 time per day
- ☐ 2 or more times per day

126a. Each time you ate **chocolate candy**, how much did you usually eat?

- ☐ Less than 1 average bar or less than 1 ounce
- ☐ 1 average bar or 1 to 2 ounces
- ☐ More than 1 average bar or more than 2 ounces

Question 127 appears in the next column

127. How often did you eat **other candy**?

- ☐ NEVER (GO TO QUESTION 128)
- ☐ 1 time in past month
- ☐ 2–3 times in past month
- ☐ 1 time per week
- ☐ 2 times per week
- ☐ 3–4 times per week
- ☐ 5–6 times per week
- ☐ 1 time per day
- ☐ 2 or more times per day

127a. Each time you ate **other candy**, how much did you usually eat?

- ☐ Fewer than 2 pieces
- ☐ 2 to 9 pieces
- ☐ More than 9 pieces

128. How often did you eat **eggs, egg whites,** or **egg substitutes** (NOT counting eggs in baked goods and desserts)? *(Please include eggs in salads, quiche, and soufflés.)*

- ☐ NEVER (GO TO QUESTION 129)
- ☐ 1 time in past month
- ☐ 2–3 times in past month
- ☐ 1 time per week
- ☐ 2 times per week
- ☐ 3–4 times per week
- ☐ 5–6 times per week
- ☐ 1 time per day
- ☐ 2 or more times per day

128a. Each time you ate **eggs**, how many did you usually eat?

- ☐ 1 egg
- ☐ 2 eggs
- ☐ 3 or more eggs

128b. How often were the eggs you ate **egg substitutes** or **egg whites only**?

- ☐ Almost never or never
- ☐ About ¼ of the time
- ☐ About ½ of the time
- ☐ About ¾ of the time
- ☐ Almost always or always

128c. How often were the eggs you ate **regular whole eggs**?

- ☐ Almost never or never
- ☐ About ¼ of the time
- ☐ About ½ of the time
- ☐ About ¾ of the time
- ☐ Almost always or always

128d. How often were the eggs you ate **cooked in oil, butter,** or **margarine**?

- ☐ Almost never or never
- ☐ About ¼ of the time
- ☐ About ½ of the time
- ☐ About ¾ of the time
- ☐ Almost always or always

Question 129 appears on the next page

This is a sample form. Do not use for scanning.

Over the past month…

128e. How often were the eggs you ate part of **egg salad**?

- ☐ Almost never or never
- ☐ About ¼ of the time
- ☐ About ½ of the time
- ☐ About ¾ of the time
- ☐ Almost always or always

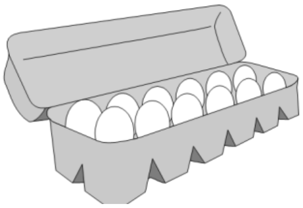

129. How many cups of **coffee**, caffeinated or decaffeinated, did you drink (including coffee drinks such as Latte, Mocha, Frappuccino, etc.)?

- ☐ NONE (GO TO QUESTION 130)

- ☐ Less than 1 cup in past month
- ☐ 1–3 cups in past month
- ☐ 1 cup per week
- ☐ 2–4 cups per week
- ☐ 5–6 cups per week
- ☐ 1 cup per day
- ☐ 2–3 cups per day
- ☐ 4–5 cups per day
- ☐ 6 or more cups per day

129a. How often was the coffee you drank **decaffeinated**?

- ☐ Almost never or never
- ☐ About ¼ of the time
- ☐ About ½ of the time
- ☐ About ¾ of the time
- ☐ Almost always or always

130. How many glasses, cans, or bottles of **COLD** or **ICED tea,** caffeinated or decaffeinated, did you drink?

- ☐ NONE (GO TO QUESTION 131)

- ☐ Less than 1 glass, can or bottle in past month
- ☐ 1–3 glasses, cans or bottles in past month
- ☐ 1 glass, can or bottle per week
- ☐ 2–4 glasses, cans or bottles per week
- ☐ 5–6 glasses, cans or bottles per week
- ☐ 1 glass, can or bottle per day
- ☐ 2–3 glasses, cans or bottles per day
- ☐ 4–5 glasses, cans or bottles per day
- ☐ 6 or more glasses, cans or bottles per day

Question 131 appears in the next column

130a. How often was the cold or iced tea you drank **decaffeinated** or **herbal**?

- ☐ Almost never or never
- ☐ About ¼ of the time
- ☐ About ½ of the time
- ☐ About ¾ of the time
- ☐ Almost always or always

130b. How often was the cold or iced tea you drank **presweetened with either sugar or artificial sweeteners** (such as Splenda, Equal, Sweet'N Low or others)?

- ☐ Almost never or never (GO TO QUESTION 131)
- ☐ About ¼ of the time
- ☐ About ½ of the time
- ☐ About ¾ of the time
- ☐ Almost always or always

130c. What kind of **sweetener** was added to your presweetened cold or iced tea most of the time?

- ☐ Sugar or honey
- ☐ Artificial sweeteners (such as Splenda, Equal, Sweet 'N Low or others)

131. How many cups of **HOT tea**, caffeinated or decaffeinated, did you drink?

- ☐ NONE (GO TO QUESTION 132)

- ☐ Less than 1 cup in past month
- ☐ 1–3 cups in past month
- ☐ 1 cup per week
- ☐ 2–4 cups per week
- ☐ 5–6 cups per week
- ☐ 1 cup per day
- ☐ 2–3 cups per day
- ☐ 4–5 cups per day
- ☐ 6 or more cups per day

131a. How often was the hot tea you drank **decaffeinated** or **herbal**?

- ☐ Almost never or never
- ☐ About ¼ of the time
- ☐ About ½ of the time
- ☐ About ¾ of the time
- ☐ Almost always or always

Question 132 appears on the next page

This is a sample form. Do not use for scanning.

Over the <u>past month</u>…

132. Over the <u>past month</u>, did you add **sugar, honey or other sweeteners** to your tea or coffee (hot or iced)?

☐ NO (GO TO QUESTION 133)
☐ YES

132a. How often did you add **sugar** or **honey** to your coffee or tea (hot or iced)?

☐ Almost never or never (GO TO QUESTION 132c)
☐ About ¼ of the time
☐ About ½ of the time
☐ About ¾ of the time
☐ Almost always or always

132b. Each time **sugar** or **honey** was added to your coffee or tea, how much was usually added?

☐ Less than 1 teaspoon
☐ 1 to 3 teaspoons
☐ More than 3 teaspoons

132c. How often did you add **artificial sweetener** (such as Splenda, Equal, Sweet'N Low or others) to your coffee or tea?

☐ Almost never or never (GO TO QUESTION 133)
☐ About ¼ of the time
☐ About ½ of the time
☐ About ¾ of the time
☐ Almost always or always

132d. What kind of **artificial sweetener** did you usually use?

☐ Equal or aspartame
☐ Sweet'N Low or saccharin
☐ Splenda or sucralose
☐ Herbal extracts or other kind

132e. Each time **artificial sweetener** was added to your coffee or tea, how much was usually added?

☐ Less than 1 packet or less than 1 teaspoon
☐ 1 packet or 1 teaspoon
☐ More than 1 packet or more than 1 teaspoon

Question 133 appears in the next column

133. Over the <u>past month</u>, did you add **whiteners** (such as cream, milk, or non-dairy creamer) to your tea or coffee?

☐ NO (GO TO QUESTION 134)
☐ YES

133a. How often was **non-dairy creamer** added to your coffee or tea?

☐ Almost never or never (GO TO QUESTION 133d)
☐ About ¼ of the time
☐ About ½ of the time
☐ About ¾ of the time
☐ Almost always or always

133b. Each time **non-dairy creamer** was added to your coffee or tea, how much was usually used?

☐ Less than 1 teaspoon
☐ 1 to 3 teaspoons
☐ More than 3 teaspoons

133c. What kind of **non-dairy creamer** did you usually use?

☐ Regular powdered
☐ Low-fat or fat-free powdered
☐ Regular liquid
☐ Low-fat or fat-free liquid

133d. How often was **cream** or **half and half** added to your coffee or tea?

☐ Almost never or never (GO TO QUESTION 133f)
☐ About ¼ of the time
☐ About ½ of the time
☐ About ¾ of the time
☐ Almost always or always

133e. Each time **cream** or **half and half** was added to your coffee or tea, how much was usually added?

☐ Less than 1 tablespoon
☐ 1 to 2 tablespoons
☐ More than 2 tablespoons

133f. How often was **milk** added to your coffee or tea?

☐ Almost never or never (GO TO QUESTION 134)
☐ About ¼ of the time
☐ About ½ of the time
☐ About ¾ of the time
☐ Almost always or always

Question 134 appears on the next page

This is a sample form. Do not use for scanning.

Over the past month…

133g. Each time **milk** was added to your coffee or tea, how much was usually added?

- ☐ Less than 1 tablespoon
- ☐ 1 to 3 tablespoons
- ☐ More than 3 tablespoons

133h. What kind of **milk** was usually added to your coffee or tea?

- ☐ Whole milk
- ☐ 2% milk
- ☐ 1% milk
- ☐ Skim, nonfat, or ½% milk
- ☐ Evaporated or condensed (canned) milk
- ☐ Soy milk
- ☐ Rice milk
- ☐ Other

134. How often was **sugar** or **honey** added to foods you ate? *(Please do not include sugar in coffee, tea, other beverages, or baked goods.)*

- ☐ NEVER (GO TO INTRODUCTION TO QUESTION 135)
- ☐ 1 time in past month
- ☐ 2–3 times in past month
- ☐ 1 time per week
- ☐ 2 times per week
- ☐ 3–4 times per week
- ☐ 5–6 times per week
- ☐ 1 time per day
- ☐ 2 or more times per day

134a. Each time **sugar** or **honey** was added to foods you ate, how much was usually added?

- ☐ Less than 1 teaspoon
- ☐ 1 to 3 teaspoons
- ☐ More than 3 teaspoons

The following questions are about the kinds of margarine, mayonnaise, sour cream, cream cheese, and salad dressing that you ate. If possible, please check the labels of these foods to help you answer.

135. Over the past month, did you eat **margarine**?

- ☐ NO (GO TO QUESTION 136)
- ☐ YES

135a. How often was the margarine you ate **light**, **low-fat**, or **fat-free** (stick or tub)?

- ☐ Almost never or never
- ☐ About ¼ of the time
- ☐ About ½ of the time
- ☐ About ¾ of the time
- ☐ Almost always or always

Question 136 appears in the next column

136. Over the past month, did you eat **butter**?

- ☐ NO (GO TO QUESTION 137)
- ☐ YES

136a. How often was the butter you ate **light** or **low-fat**?

- ☐ Almost never or never
- ☐ About ¼ of the time
- ☐ About ½ of the time
- ☐ About ¾ of the time
- ☐ Almost always or always

137. Over the past month, did you eat **mayonnaise** or **mayonnaise-type dressing**?

- ☐ NO (GO TO QUESTION 138)
- ☐ YES

137a. How often was the mayonnaise you ate **light, low-fat or fat-free**?

- ☐ Almost never or never
- ☐ About ¼ of the time
- ☐ About ½ of the time
- ☐ About ¾ of the time
- ☐ Almost always or always

138. Over the past month, did you eat **sour cream**?

- ☐ NO (GO TO QUESTION 139)
- ☐ YES

138a. How often was the sour cream you ate **light, low-fat,** or **fat-free**?

- ☐ Almost never or never
- ☐ About ¼ of the time
- ☐ About ½ of the time
- ☐ About ¾ of the time
- ☐ Almost always or always

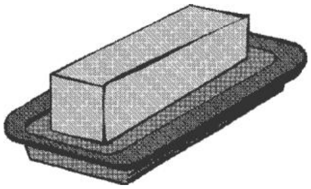

Question 139 appears on the next page

This is a sample form. Do not use for scanning.

Over the past month…

139. Over the past month, did you eat **cream cheese**?

☐ NO (GO TO QUESTION 140)

☐ YES

139a. How often was the cream cheese you ate **light, low-fat,** or **fat-free**?

☐ Almost never or never
☐ About ¼ of the time
☐ About ½ of the time
☐ About ¾ of the time
☐ Almost always or always

140. Over the past month, did you eat **salad dressing**?

☐ NO (GO TO INTRODUCTION TO QUESTION 141)

☐ YES

140a. How often was the salad dressing you ate **light,** low-**fat** or **fat-free**?

☐ Almost never or never
☐ About ¼ of the time
☐ About ½ of the time
☐ About ¾ of the time
☐ Almost always or always

The following two questions ask you to summarize your usual intake of vegetables and fruits. Please do not include salads, potatoes, or juices.

141. Over the past month, how many servings of **vegetables** (not including salad or potatoes) did you eat per week or per day?

☐ Less than 1 per week
☐ 1–2 per week
☐ 3–4 per week
☐ 5–6 per week
☐ 1 per day
☐ 2 per day
☐ 3 per day
☐ 4 per day
☐ 5 or more per day

142. Over the past month, how many servings of **fruit** (not including juices) did you eat per week or per day?

☐ Less than 1 per week
☐ 1–2 per week
☐ 3–4 per week
☐ 5–6 per week
☐ 1 per day
☐ 2 per day
☐ 3 per day
☐ 4 per day
☐ 5 or more per day

143. Over the past month, which of the following foods did you eat **AT LEAST THREE TIMES?** ***(Mark all that apply.)***

☐ Avocado, guacamole
☐ Cheesecake
☐ Chocolate, fudge, or butterscotch toppings or syrups
☐ Chow mein noodles
☐ Croissants
☐ Dried apricots
☐ Egg rolls
☐ Granola bars
☐ Hot peppers
☐ Jell-O, gelatin
☐ Mangoes
☐ Milkshakes or ice-cream sodas
☐ Olives
☐ Oysters
☐ Pickles or pickled vegetables or fruit
☐ Plantains
☐ Pork neck bones, hock, head, feet
☐ Pudding or custard
☐ Veal, venison, lamb
☐ Whipped cream, regular
☐ Whipped cream, substitute
☐ NONE

144. For **ALL** of the past month, have you followed any type of **vegetarian diet**?

☐ NO (GO TO INTRODUCTION TO QUESTION 145)

☐ YES

144a. Which of the following foods did you **TOTALLY EXCLUDE** from your diet? ***(Mark all that apply.)***

☐ Meat (beef, pork, lamb, etc.)
☐ Poultry (chicken, turkey, duck)
☐ Fish and seafood
☐ Eggs
☐ Dairy products (milk, cheese, etc.)

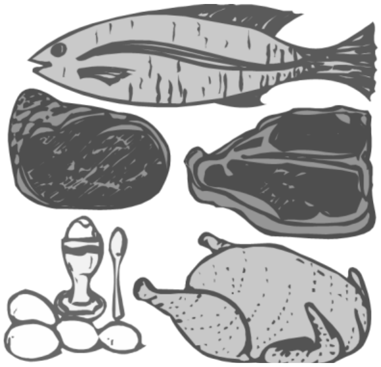

Introduction to Question 145 appears on the next page

This is a sample form. Do not use for scanning.

The next questions are about your use of vitamin pills or other supplements.

145. Over the past month, did you take any **multivitamins**, such as One-a-Day-, Theragran-, Centrum-, or Prenatal-type multivitamins (as pills, liquids, or packets)?

- ☐ NO (GO TO INTRODUCTION TO QUESTION 147)
- ☐ YES

146. How often did you take **One-a-day-, Theragran-, Centrum- or Prenatal-type** multivitamins?

- ☐ 1–3 days in past month
- ☐ 1–3 days per week
- ☐ 4–6 days per week
- ☐ Every day

146a. Did your **multivitamin** usually contain **minerals** (such as iron, zinc, etc.)?

- ☐ NO
- ☐ YES
- ☐ Don't know

146b. Over the past month, did you take any **vitamins**, **minerals**, or **other herbal supplements** other than your multivitamin?

- ☐ NO

Thank you *very much* for completing this questionnaire! Because we want to be able to use all the information you have provided, we would greatly appreciate it if you would please take a moment to review each page making sure that you:

- **Did not skip any pages and**
- **Crossed out the incorrect answer and circled the correct answer if you made any changes.**

- ☐ YES (GO TO INTRODUCTION TO QUESTION 147

Introduction to Question 147 appears in the next column

These last questions are about the vitamins, minerals, or herbal supplements you took that are NOT part of a One-a-day-, Theragran-, or Centrum-type of multivitamin.

Over the past month…

147. How often did you take **Antacids such as Tums or Rolaids**?

- ☐ NEVER (GO TO QUESTION 148)
- ☐ 1–3 days per month
- ☐ 1–3 days per week
- ☐ 4–6 days per week
- ☐ Every day

147a. When you took **Antacids such as Tums or Rolaids**, about how many tablets or lozenges did you take in one day?

- ☐ Less than 1
- ☐ 1
- ☐ 2
- ☐ 3
- ☐ 4 or more
- ☐ Don't know

147b. Was your antacid usually “extra strength”?

- ☐ NO
- ☐ YES
- ☐ Don't know

148. How often did you take **Calcium** (with or without Vitamin D) (**NOT** as part of a multivitamin in Question 146 or antacid in Question 147)?

- ☐ NEVER (GO TO QUESTION 149)
- ☐ 1–3 days per month
- ☐ 1–3 days per week
- ☐ 4–6 days per week
- ☐ Every day

148a. When you took **Calcium** , about how much elemental calcium did you take in one day? *(If possible, please check the label for elemental calcium.)*

- ☐ Less than 500 mg
- ☐ 500–599 mg
- ☐ 600–999 mg
- ☐ 1,000 mg or more
- ☐ Don't know

148b. Did your **Calcium** usually contain **Vitamin D**?

- ☐ NO
- ☐ YES
- ☐ Don't know

Question 149 appears on the next page

This is a sample form. Do not use for scanning.

Over the past month…

148c. Did your **Calcium** usually contain **Magnesium**?

☐ NO
☐ YES
☐ Don't know

148d. Did your **Calcium** usually contain **Zinc**?

☐ NO
☐ YES
☐ Don't know

149. How often did you take **Iron** (**NOT** as part of a multivitamin in Question 146)?

☐ NEVER

☐ 1–3 days per month
☐ 1–3 days per week
☐ 4–6 days per week
☐ Every day

150. How often did you take **Vitamin C** (**NOT** as part of a multivitamin in Question 146)?

☐ NEVER (GO TO QUESTION 151)

☐ 1–3 days per month
☐ 1–3 days per week
☐ 4–6 days per week
☐ Every day

150a. When you took **Vitamin C**, about how much did you take in one day?

☐ Less than 500 mg
☐ 500–999 mg
☐ 1,000–1,499 mg
☐ 1,500–1,999 mg
☐ 2,000 mg or more
☐ Don't know

151. How often did you take **Vitamin E** (**NOT** as part of a multivitamin in Question 146)?

☐ NEVER (GO TO INTRODUCTION TO QUESTION 152)

☐ 1–3 days per month
☐ 1–3 days per week
☐ 4–6 days per week
☐ Every day

Introduction to Question 152 appears in the next column

151a. When you took **Vitamin E**, about how much did you take in one day?

☐ Less than 400 IU
☐ 400–799 IU
☐ 800–999 IU
☐ 1,000 IU or more
☐ Don't know

The last two questions ask you about other supplements you took more than once per week.

152. Please mark any of the following **single supplements** you took more than once per week (**NOT** as part of a multivitamin in Question 147):

☐ B-6
☐ B-complex
☐ B-12
☐ Beta-carotene
☐ Folic acid/folate
☐ Magnesium
☐ Occu-vite/Eye health
☐ Potassium
☐ Selenium
☐ Vitamin A
☐ Vitamin D
☐ Zinc

153. Please mark any of the following **herbal, botanical, or other supplements** you took more than once per week.

☐ Chondroitin
☐ Coenzyme Q-10
☐ Echinacea
☐ Energy supplements
☐ Fish oil/omega 3's
☐ Flaxseed/oil
☐ Garlic
☐ Ginger
☐ Ginkgo biloba
☐ Ginseng
☐ Glucosamine/ chondroitin
☐ Peppermint
☐ Probiotics
☐ Saw palmetto
☐ Soy supplement
☐ Sports supplements
☐ St. John's wort
☐ Other

Thank you *very much* for completing this questionnaire! Because we want to be able to use all the information you have provided, we would greatly appreciate it if you would please take a moment to review each page making sure that you:

- **Did not skip any pages and**
- **Crossed out the incorrect answer and circled the correct answer if you made any changes.**
